# Mechanistic insights into the loss-of-function mechanisms of rare human D-amino acid oxidase variants implicated in amyotrophic lateral sclerosis

**DOI:** 10.1038/s41598-020-74048-2

**Published:** 2020-10-13

**Authors:** Aditya K. Padhi, Kam Y. J. Zhang

**Affiliations:** Laboratory for Structural Bioinformatics, Center for Biosystems Dynamics Research, RIKEN, 1-7-22 Suehiro, Tsurumi, Yokohama, Kanagawa 230-0045 Japan

**Keywords:** Protein function predictions, Predictive medicine

## Abstract

Impaired enzymatic activity in D-amino acid oxidase (DAAO) caused by missense mutations has been shown to trigger amyotrophic lateral sclerosis (ALS) through an abnormal accumulation of D-serine in the spinal cord. While loss of enzymatic functions of certain ALS-causing DAAO variants have been studied before, a detailed understanding of structure-dynamics-function relationship of the rare DAAO variants has not been investigated hitherto. To address this, we carried out a comprehensive study of all the reported rare DAAO variants. By employing a spectrum of bioinformatics analyses along with extensive structural dynamics simulations, we show that certain rare variants disrupted key interactions with the active site and decreased the conformational flexibility of active site loop comprising residues 216–228, which is essential for substrate binding and product release. Moreover, these variants lost crucial interactions with the cofactor flavin-adenine-dinucleotide, resulting in weaker binding affinity. A detailed inspection revealed that these variants exhibited such characteristics due to the abrogation of specific salt bridges. Taken together, our study provides a gateway into the structural-dynamic features of the rare DAAO variants and highlights the importance of informatics-based integrated analyses in the screening and prioritization of variants a priori to the clinical-functional characterization.

## Introduction

ALS, also known as motor neurone disease, is a fatal and progressive neurodegenerative disease that causes the death of neurons controlling voluntary muscles and eventually leads to paralysis and death of patients within 3–5 years of disease onset^1^. The lack of a treatment or disease halting therapy makes the affected individuals extremely vulnerable. While 5–10% of all ALS cases are associated with a family history, remaining 90–95% of them occur sporadically but having identical clinical features^2^. Genetic factors contribute to both familial ALS (fALS) and sporadic ALS (sALS) and their interplay is extremely complex. So far, more than 30 genes have been attributed for ALS and in excess of 100 genes have been either associated with disease risk or increased susceptibility^3,4^. Several pathological mechanisms and processes including protein misfolding, aberrant RNA processing, DNA repair, mitochondrial dysfunction, oxidative stress, neuroinflammation and axonal transport are often affected by these genetic variants and lead to ALS manifestation^5^. While a considerable progress has been made in the past decades to understand the genetic basis of ALS, there remains an enormous challenge in characterizing these genetic variants because of their excessive number and nearly impossible task to carry out functionally relevant experiments^6,7^. Moreover, due to the advancement of genome wide association studies (GWAS) and whole exome sequencing studies, the number of genetic variants including the rare variants, have grown colossally in recent years^8–11^. These variants are associated with both fALS and sALS, and thus cause an increased burden in ALS patients. Recent studies further indicate that these rare variants, such as the low-frequency ones with minor allele frequency (MAF) of 1–5% or modest ones with MAF < 1%, or an amalgamation of both could fill the missing genetic basis of ALS, undetermined so far.

In this milieu, it is extremely important to understand the functional consequences and disease predisposition capabilities of the rare variants in order to define the genetic paradigm of ALS. As rare variants are known to cause an increased disease risk across diverse populations, not only their discovery but also understanding their structure–function relationship is warranted in order to comprehend the origin and progression of ALS. In light of this observation, we focused our study on the rare variants catalogued in the Project MinE consortium, whose goal is to sequence the whole genome of > 15,000 ALS patients and ~ 7500 control individuals to provide a comprehensive genetic architecture of ALS^12^.

**Figure 1:**
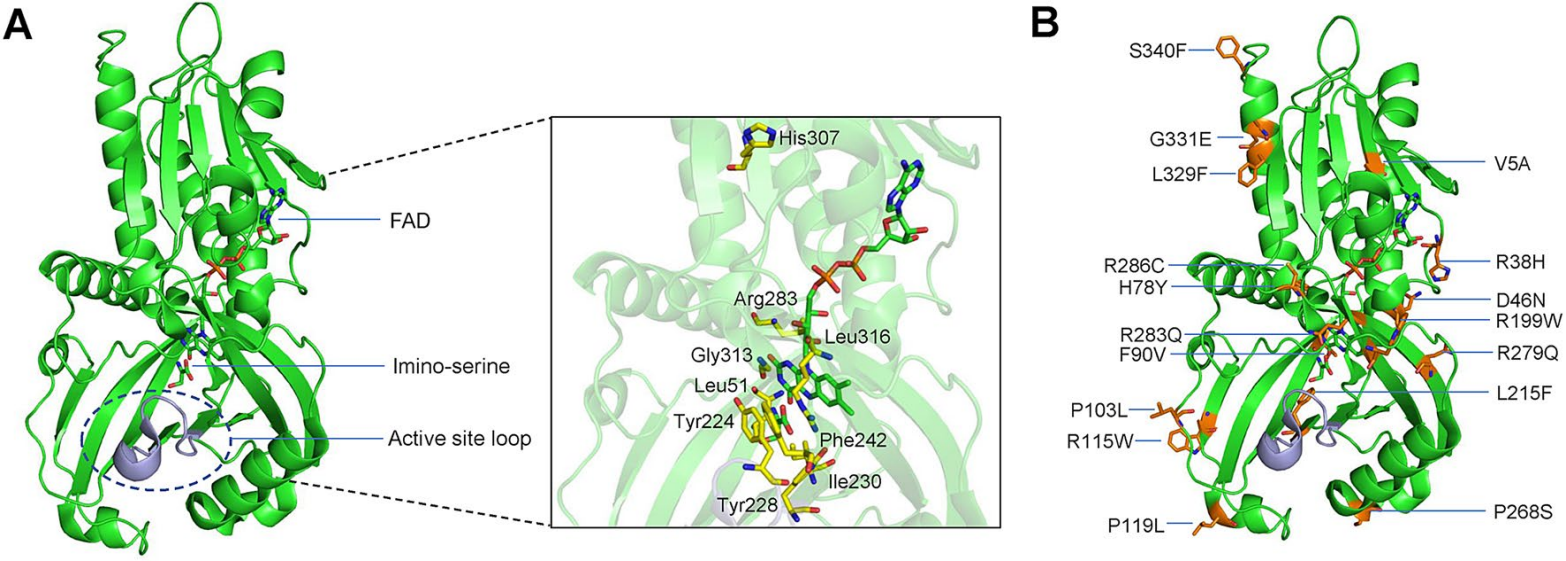
Graphic representation of human D-amino acid oxidase showing its functional sites and rare DAAO variants. (**A**) Diagrammatic representation of the structure of human D-amino acid oxidase showing its functional sites. Cofactor FAD and product imino-serine are shown as stick models, active site loop comprising residues 216–228 is shown in gray color, and active site residues are shown as yellow stick models in the expanded section. (**B**) All the rare variants of DAAO are labeled and represented as stick models in orange color. Figures are generated using the PyMOL Molecular Graphics System, Version 1.5.0.4, Schrödinger, LLC.

To achieve this, we mined the Project MinE Variant Browser for DAAO variants owing to its increased association with ALS. This led to the identification of 20 rare variants from different populations, among which three were previously studied^13–15^. Human DAAO, a flavin-adenine-dinucleotide (FAD)-dependent oxidase, governs its neuroprotective functions via its active site residues, in addition to an active site loop comprising residues 216 to 228 that acts as an “active site lid”, transitioning between a “closed” state observed in crystal structures and an “open” state required for the binding of substrate and release of product (Fig. 1A)^16^. These structural features facilitate in the catalysis of D-alanine, D-serine, and D-proline to their corresponding keto-acids in human DAAO through the process of oxidative deamination^17^. Several interesting studies using biochemical and functional characterization have shown that (1) mutations in Arg120 in human DAAO favor FAD-binding and nuclear mistargeting, (2) the G183R-DAAO variant loses its ability to degrade D-serine, leading to the formation of protein aggregates and is partially targeted to peroxisomes at cellular level, (3) FAD affinity is significantly increased by the presence of a ligand in the DAAO active sites, and (4) some variants are potentially susceptible to an increased risk of schizophrenia^18–21^. Moreover, the R199W and R199Q DAAO variants identified from ALS patients caused an altered protein conformation, resulting in loss of enzymatic activity and abnormal D-serine levels^22^. The loss of enzymatic activity, and excessive accumulation of D-serine in the spinal cord was further observed in sALS patients and in SOD1^G93A^-ALS mouse model^14^. Because compromised enzymatic activity of DAAO happens to play a vital role in manifestation of ALS, as a result of excessive accumulation of D-serine, we recently demonstrated that certain structural and dynamic changes disrupts the enzymatic activity in ALS associated DAAO variants, leading to the disease causation^23^. This encouraged us to investigate if any of the Project MinE derived rare DAAO variants could also exhibit loss of enzymatic functions and if so, how and by what molecular mechanisms. To achieve this, we carried out several bioinformatics analyses in conjunction with extensive all-atom molecular dynamics (MD) simulations for wild-type (WT) and fifteen rare DAAO variants. All the DAAO rare variants with their corresponding ID and allele frequencies are presented in Table 1 and shown in Fig. 1B. Our integrated study including MD simulations and in silico approaches reveal that certain structural and dynamic attributes corresponding to the active site residues, an active site loop comprising residues 216–228 and binding with cofactor FAD, contribute to the plausible loss of enzymatic activity, thus likely to be predisposing the individuals carrying these variations to ALS. This report and our earlier study^23^, employing MD simulations and other informatics-based analyses clearly demonstrate that certain Project MinE based rare DAAO variants are also loss-of-function type and could cause ALS through an unusual deposition of D-serine in the spinal cord.

**Table 1:** Rare variants of DAAO retrieved from ProjectMinE and analyzed in this study.

DAAO rare variants	ID	Allele frequency (cases)*	Allele frequency (controls)*	Allele frequency (all)*
V5A	chr12:109,278,796:T:C	0.000115	0	0.0000807
R38H^#^	chr12:109,278,895:G:A;rs368093324	0.000115	0	0.0000807
D46N	chr12:109,278,918:G:A;rs375063129	0.000115	0	0.0000807
H78Y	chr12:109,281,263:C:T;rs374188691	0.000115	0	0.0000807
F90V	chr12:109,281,299:T:G;rs146917361	0.000344	0	0.000242
P103L	chr12:109,281,339:C:T;rs200127576	0.000115	0	0.0000807
R115W	chr12:109,283,278:C:T;rs201583577	0.000573	0.000819	0.000645
P119L	chr12:109,283,291:C:T;rs780068280	0.000115	0	0.0000807
R199W^#^	chr12:109,288,126:C:T;rs139166976	0.000229	0.000273	0.000242
R199Q^#^	chr12:109,288,127:G:A;rs200850756	0.000229	0	0.000161
L215F	chr12:109,290,812:C:T;rs369202408	0.000115	0	0.0000807
P268S	chr12:109,292,561:C:T;rs202065454	0.000229	0	0.000161
R279Q	chr12:109,293,175:G:A;rs888461072	0.000115	0	0.0000807
R283Q	chr12:109,293,187:G:A;rs143550642	0.000344	0	0.000242
R286C	chr12:109,293,195:C:T;rs768403852	0.000115	0	0.0000807
L329F	chr12:109,294,252:C:T;rs762733390	0.000115	0	0.0000807
G331E	chr12:109,294,259:G:A;rs4262766	0.000229	0.000273	0.000242
S340F	chr12:109,294,286:C:T;rs376435571	0.000115	0	0.0000807
S345F	chr12:109,294,301:C:T;rs143732132	0.000344	0.000273	0.000323
S345C	chr12:109,294,301:C:G	0.000115	0	0.0000807

*Allele frequency (cases): Allele Frequency among all Project MinE cases, Allele frequency (controls): Allele Frequency among all Project MinE controls, Allele frequency (all): Allele Frequency among all Project MinE cases and controls, #Variants not included in the present study.

## Results and discussion

In this study, we carried out a comprehensive analysis of all the Project MinE catalogued DAAO rare variants to examine the structural and dynamic changes related to the enzymatic activity and subsequently ALS association.

### DAAO native residues and their evolutionary preservation

Evolutionary conservation profiles of all the reviewed DAAO sequences from UniprotKB across 14 species showed that R199, P268, R283 and R286 residues were completely conserved whereas V5, R38, D46, P103, R115, P119, L329, G331 and S345 residues were present in more than 50% the species (Figure S1). This suggests that certain rare variants such as R199W, P268S, R283Q and R286C that alter the WT-DAAO residues might be pathogenic and may negatively influence the structure and function of DAAO, eventually predisposing in disease etiology and progression.

### MD simulation based structural and dynamic analysis

#### Structural stability, flexibility, compactness and secondary structure of DAAO rare variants

The structural and dynamic features of the rare variants were analysed to examine if they exhibit any changes in the functionally important residues that are all necessary for the proper functioning of DAAO and its enzymatic activity.

**Figure 2:**
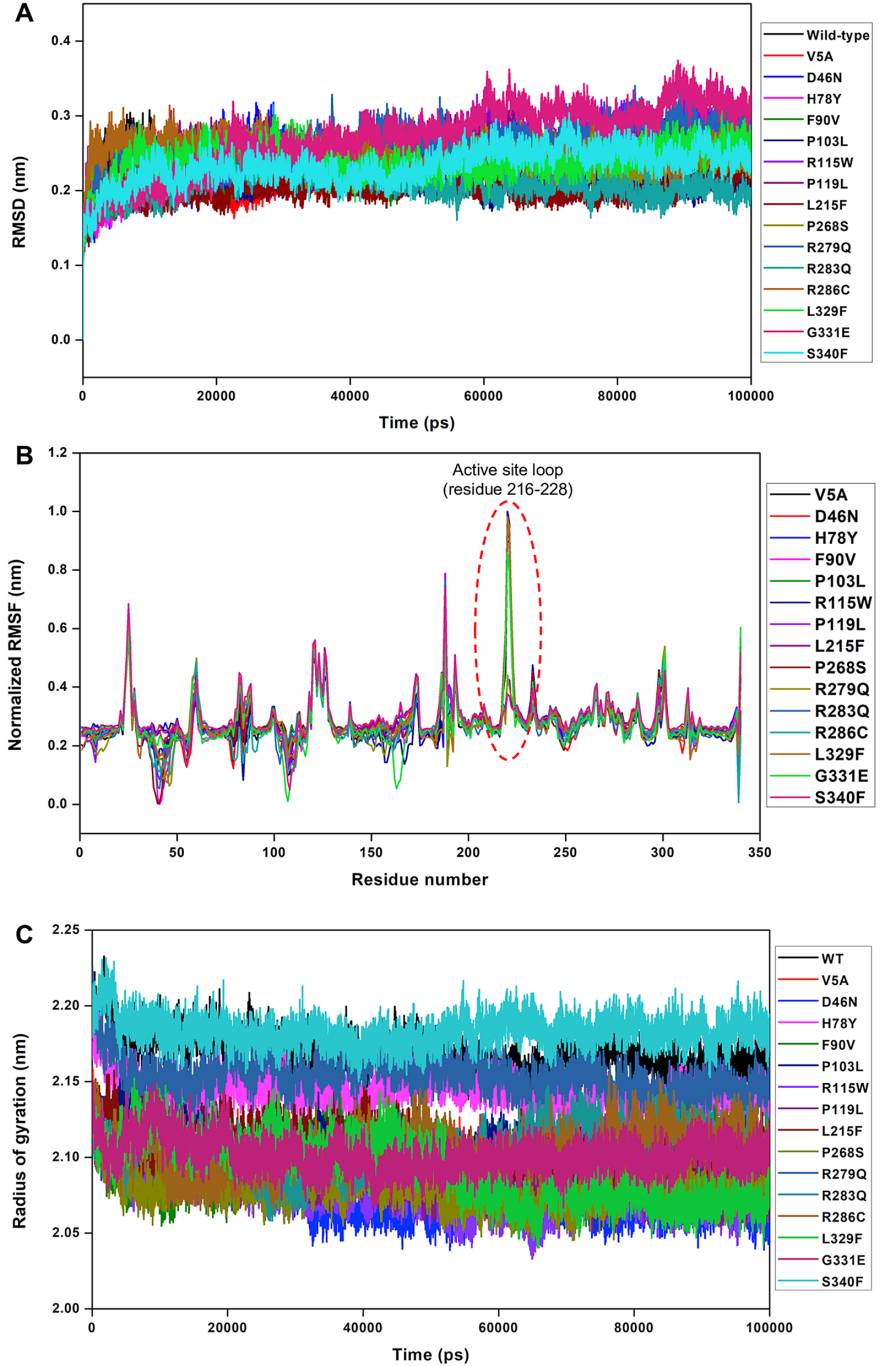
Root mean square deviation (RMSD), root mean square fluctuation (RMSF) and radius of gyration (Rg) profiles of DAAO rare variants. (**A**) Plots showing the backbone RMSD profiles of WT and rare DAAO variants during the course of MD simulations, (**B**) RMSF values of atomic positions computed for the backbone atoms of rare DAAO variants are shown as a function of residue number, where RMSF of each variant is subtracted from the WT. The higher RMSF value at around active site loop residues 216 to 228, indicates that the WT and certain variants experience more fluctuation and, thus, higher structural flexibility during simulations, (**C**) Radius of gyration of C⍺ atoms for WT and DAAO variants as a function of time at 300 K is shown.

First, the overall structural stability and convergence of the rare variants were examined by computing the backbone RMSDs and it was found that all the variants exhibited a very identical deviation to that of WT (Fig. 2A, Figure S2). Only the G331E variant, experienced a slightly increased deviation at around 60 ns and 80 ns respectively. However, as the RMSDs for all the systems were stable after 70 ns, final 30 ns trajectories were considered for most of the analysis related to structural and dynamic properties (Fig. 2A).

Second, the RMSF of each variant was computed and compared to WT-DAAO. Computed RMSF that represents the per-residue structural flexibility of the variants, showed that all the variants except H78Y, R279Q and S340F experienced a similar RMSF to that of WT-DAAO except around the active site loop encompassing residues 216–228 (Fig. 2B). While WT-DAAO exhibited more than 0.7 nm fluctuations near the active site loop consisting of residues 216–228, certain rare variants including V5A, D46N, F90V, P103L, R115W, P119L, L215F, P268S, R283Q, R286C, L329F and G331E significantly reduced fluctuations around the active site loop residues and close to the hydrophobic stretch region of residues 47–51. Overall, the mean and standard deviations of the computed RMSFs reflect this behaviour during the simulations.

Third, the impact of the rare variants on the compactness of the proteins during the MD simulations were examined by computing the radius of gyration (Fig. 2C, Figure S3). It was found that while V5A, D46N, F90V, P103L, R115W, P119L, L215F, P268S, R283Q, R286C, L329F and G331E variants exhibited nearly similar radius of gyration, the H78Y, R279Q and S340F variants including the WT-DAAO exhibited a slightly increased profile. Overall, it was found that V5A, D46N, F90V, P103L, R115W, P119L, L215F, P268S, R283Q, R286C, L329F and G331E rare DAAO variants experienced more compact structure than WT and H78Y, R279Q and S340F during the course of simulations, supporting the RMSF characteristic of reduced conformational flexibility (Fig. 2C, Figure S3).

Fourth, the role of mutations on local structure stability of the rare variants were analyzed by computing the secondary structural elements during simulations. It was found that substitutions in some of the rare variants, such as, (1) in D46N, caused helix to turn during the simulations, (2) the R115W substitution while retained the sheet for maximum duration, also induced turn at certain instances, and (3) the P119L induced sheet conformation. These results indicated that the variants did not significantly impact the secondary structure during the simulations and the overall stability of the variants, further correlating with the RMSD profiles (Figure S4).

#### Dynamics and plasticity of the active site loop

**Figure 3:**
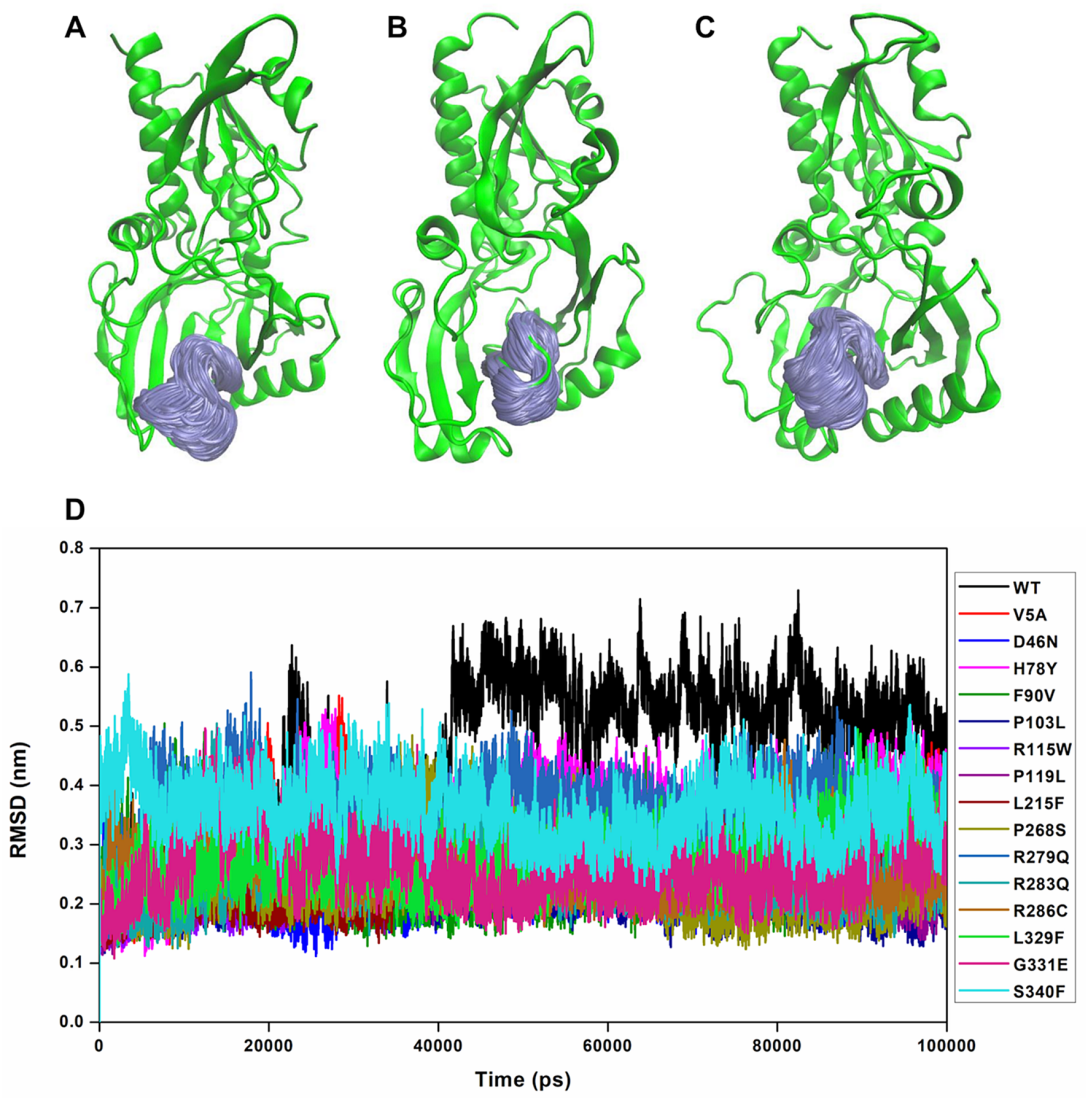
Representative figures depicting the dynamics of active site loop. Diagrammatic representation structures of (**A**) WT-DAAO, and rare DAAO variants (B and C) exhibiting conformational sampling of active site loop comprising 216–228 residues. Active site loop is shown in gray color sampled at every 100^th^ time frame, where in panels (**A**) and (**C**) a much wider range of conformational sampling of the active site loop can be seen as compared to panel (**B**), where a restricted loop flexibility was observed, (**D**) For each DAAO variant, time evolution RMSD profiles of the active site loop are presented. Figures are generated using the PyMOL Molecular Graphics System, Version 1.5.0.4, Schrödinger, LLC.

**Figure 4:**
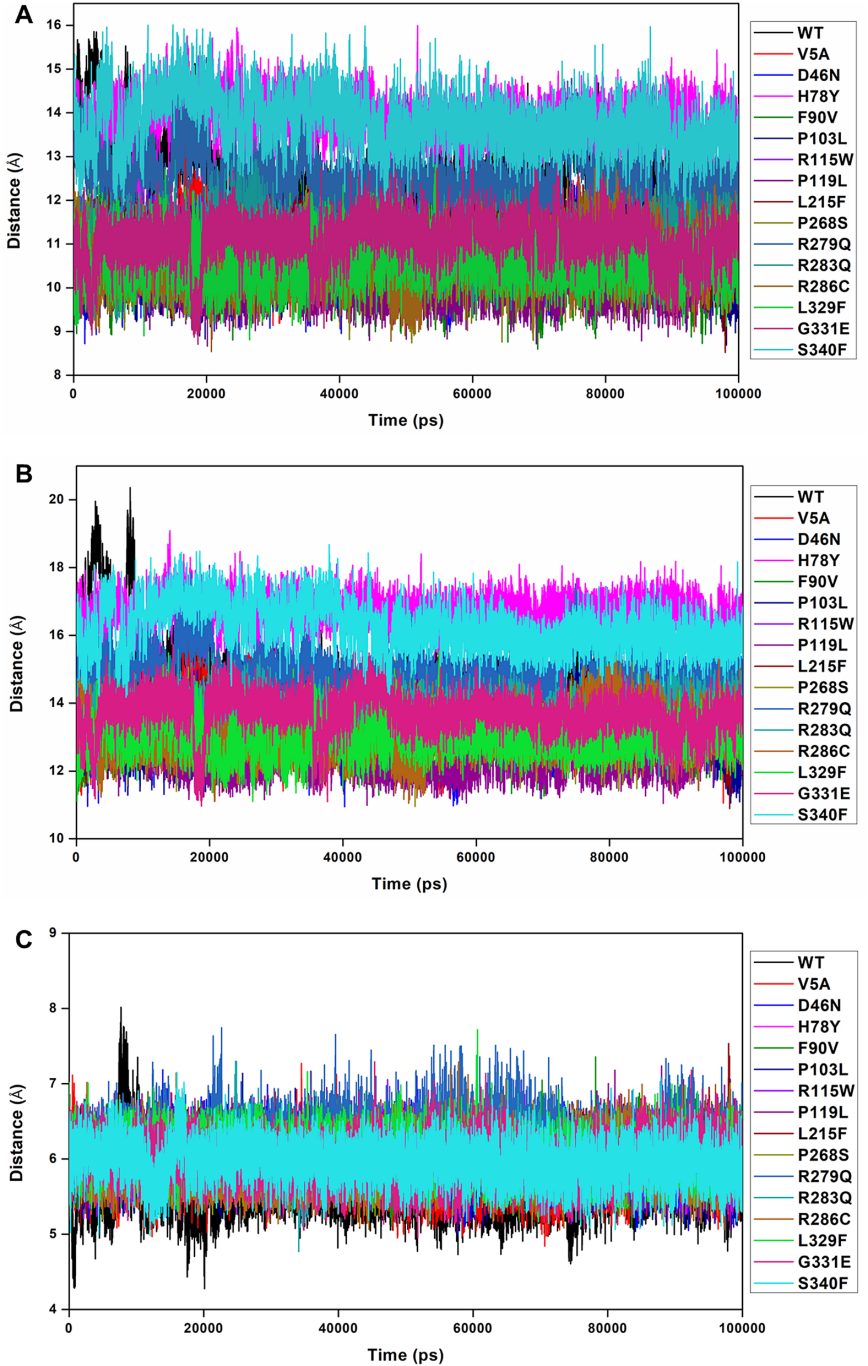
Distances between key active site loop residue pairs in DAAO rare variants. Interatomic distances between the C⍺-atoms of (**A**) Thr216-Arg221, (**B**) Arg221-Tyr228 and (**C**) Thr216-Tyr228 residues for WT and rare DAAO variants as a function of simulation time are shown.

As only the WT and H78Y, R279Q and S340F variants exhibited an increased fluctuation and conformational flexibility at around the active site loop consisting of residues 216–228, we wanted to predict the importance of such characteristics in terms of DAAO’s enzymatic activity. It is already established that active site loop consisting of residues 216–228 act as a lid in DAAO, and this transitions between a “closed” and “open” state for executing its enzymatic activity. In other words, the transition between “closed” to “open” conformation facilitates the enzymatic reaction when the substrate comes in and the product releases. Upon structural superposition of one of the open-conformation active site loop of WT-DAAO crystal structure in the presence of an inhibitor [3‐(7‐hydroxy‐2‐oxo‐4‐phenyl‐2H‐chromen‐6‐yl) propanoic acid] (PDB ID: 4QFD), onto the simulated structures of WT, it was observed that they exhibit the open state active site loop residues on top of the profusely found closed loop structures. Interestingly, it was also observed that certain rare DAAO variants including V5A, D46N, F90V, P103L, R115W, P119L, L215F, P268S, R283Q, R286C, L329F and G331E predominantly exhibited the active site loop in its closed state during the simulations (Fig. 3B). On the other hand, the H78Y, R279Q and S340F variants mainly displayed the open state active site loop conformation (Fig. 3C), similar to that of WT-DAAO (Fig. 3A). Furthermore, RMSD profiles of the active site loop residues from 216 to 228 show that the WT exhibits higher deviation during the 100 ns simulations as compared to certain other rare variants. Average RMSDs calculated from the respective trajectories further show that H78Y, R279Q and S340F variants exhibit relatively higher deviation than V5A, D46N, F90V, P103L, R115W, P119L, L215F, P268S, R283Q, R286C, L329F and G331E variants (Fig. 3D, Figure S5). Furthermore, the dynamics of the active site loop was characterized by measuring the distance of the key active site loop residues, Thr216-Arg221, Arg221-Tyr228 and Thr216-Tyr228. The calculated distance between Thr216-Arg221 and Arg221-Tyr228 pairs of residues for WT and the rare variants indicated that V5A, D46N, F90V, P103L, R115W, P119L, L215F, P268S, R283Q, R286C, L329F and G331E variants exhibited a considerable reduced distance as compared to WT and H78Y, R279Q and S340F variants (Fig. 4A,B). The WT and rare variants did not exhibit a major change in distance between Thr216-Tyr228 residue pairs (Fig. 4C). This gave us an impression that perhaps due to the “closed” active site loop conformation, V5A, D46N, F90V, P103L, R115W, P119L, L215F, P268S, R283Q, R286C, L329F and G331E rare variants cannot accomplish their enzymatic activity in catalyzing the oxidative deamination of D‐amino acids (primarily D-serine) and hence trigger its excessive accumulation in ALS spinal cord. This finding was interesting as early experimental reports have shown that loss of enzymatic activity in ALS causing DAAO variant R199W is an important pathogenic factor for the build-up of D-serine, a co-agonist of the NMDA glutamate receptor inducing glutamate transmission^14^. Our present results in addition to earlier reports^23^, thus indicates that this loss of enzymatic activity in DAAO variants could be originated due to “closed” conformation of active site loop, owing to which they fail to catalyze the D-serine resulting in its abnormal build-up.

#### Role of crucial salt bridges in active site loop dynamics

As certain rare DAAO variants were proposed to exhibit restricted active site loop flexibility with predominantly experiencing closed conformation during the course of simulations, we were curious to know what are the driving forces of such behaviour. During our investigation of the structural features, it was found that variants V5A, D46N, F90V, P103L, R115W, P119L, L215F, P268S, R283Q, R286C, L329F and G331E established fewer salt bridges during the simulations as compared to WT and H78Y, R279Q and S340F variants, thus restricting their active site loop flexibility and movement. Computation of salt bridges revealed that WT and H78Y, R279Q and S340F variants had overall higher number of salt bridge interactions than V5A, D46N, F90V, P103L, R115W, P119L, L215F, P268S, R283Q, R286C, L329F and G331E variants during the MD simulations. Our focused analysis on only the salt bridges that are formed by or between the active site loop residues further revealed that WT established ten salt bridges whereas the rare variants D46N and L215F exhibiting closed active site loop dynamics established only three and four interactions respectively (Table 2). Interestingly, R279Q and S340F variants formed relatively higher i.e. eight salt bridges, of which some of the unique interactions were absent in D46N and L215F variants (Table 2). The time profile salt bridge formation/dissolution, and percentage occupancy analysis for the rare DAAO variants further demonstrates this and highlight that salt bridges indeed play a major role in DAAO dynamics and eventually in the reduced conformational plasticity of active site loop in DAAO variants as compared to WT and H78Y, R279Q and S340F variants (Table 2). For clarity of representation, salt bridge interactions with respect to time for other variants have been presented in Figure S6. Overall, a thorough inspection of the MD simulation trajectories emphasized that these unique salt bridges in WT and H78Y, R279Q and S340F variants facilitate the active site loop to attain higher flexibility and transition between “closed” and “open” states as compared to the other DAAO variants preferring the “closed” state.

**Table 2:** Salt bridge interactions and their percentage occupancy for WT and rare DAAO variants from respective MD trajectories.

S. no	WT	D46N	L215F	R279Q	S340F
1	Asp218-Arg221 (5%)	Asp218-Arg221 (6%)	Asp218-Arg221 (30%)	Asp218-Arg221 (34%)	Asp218-Arg221 (11%)
2	Asp218-His99 (11%)	Absent	Asp218-His99 (0.3%)	Asp218-His99 (0.2%)	Asp218-His99 (2%)
3	Glu220-Arg221 (21%)	Glu220-Arg221 (0.2%)	Glu220-Arg221 (6%)	Glu220-Arg221 (3%)	Glu220-Arg221 (16%)
4	Asp127-Arg221 (11%)	Absent	Absent	Asp127-Arg221 (39%)	Asp127-Arg221 (0.2%)
5	Arg265-Glu261 (0.3%)	Absent	Absent	Absent	Absent
6	Asp218-Arg265 (10%)	Absent	Absent	Absent	Absent
7	Glu220-Arg265 (16%)	Absent	Absent	Glu220-Arg265 (35%)	Glu220-Arg265 (0.4%)
8	Glu100-His217 (5%)	Absent	Absent	Glu100-His217 (0.2%)	Glu100-His217 (2%)
9	Glu261-Arg221 (2%)	Absent	Absent	Glu261-Arg221 (0.2%)	Glu261-Arg221 (9%)
10	Asp255-Arg221 (75%)	Asp255-Arg221 (29%)	Asp255-Arg221 (7%)	Asp255-Arg221 (73%)	Asp255-Arg221 (23%)

### Essential dynamics and free energy landscape of DAAO rare variants

**Figure 5:**
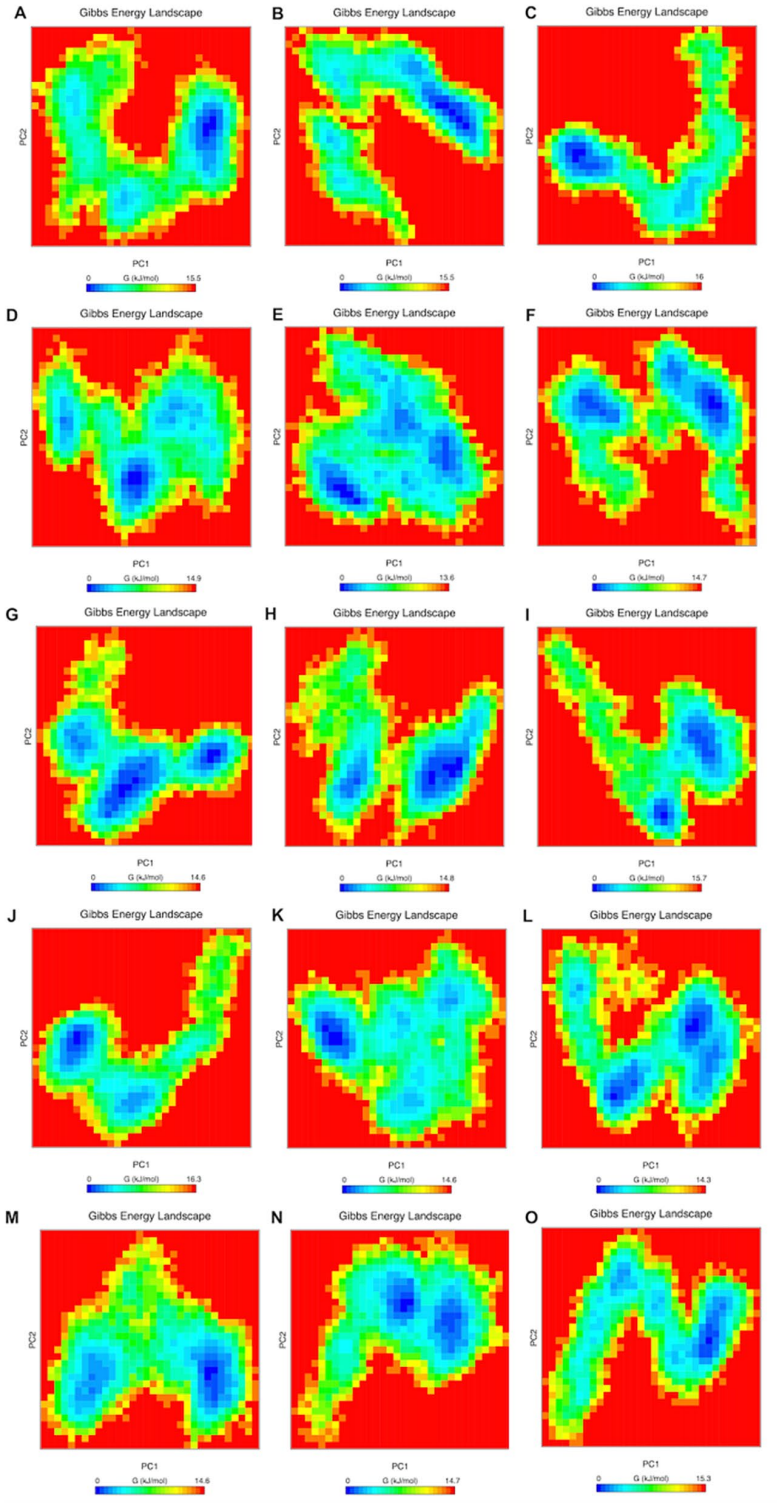
Free energy landscapes of rare DAAO variants generated by projecting the principal components, PC1 and PC2. Free energy landscapes generated by projecting the principal components, PC1 and PC2, of DAAO rare variants from MD simulations at 300 K. The free energies are represented by -*k*_B_*T* ln *P*_(PC1, PC2)_ with *P*_(PC1,PC2)_ being the distribution probability calculated using the structures sampled at 300 K. The unit of free energy is *k*_B_*T*, where *T* is the temperature, and *k*_B_ is the Boltzmann constant. FEL for variants V5A, D46N, H78Y, F90V, P103L, R115W, P119L, L215F, P268S, R279Q, R283Q, R286C, L329F, G331E and S340F are represented as (**A**–**O**).

The principal component analysis is typically used to extrapolate significant motions of proteins during MD simulations, where the essential space driven by leading eigenvalues indicate where most of the protein dynamics occur. We visualized and analyzed the clusters of stable states of PCA for WT-DAAO and all the rare variants and found that the WT and H78Y, R279Q and S340F rare variants covered a larger region of the phase space in comparison to V5A, D46N, F90V, P103L, R115W, P119L, L215F, P268S, R283Q, R286C, L329F and G331E variants during the MD simulations. From the covariance matrix, it was found that while the trace value of H78Y, R279Q and S340F variants was 19.90 nm^2^, 20.0 nm^2^ and 19.42 nm^2^, it was 17.19 nm^2^, 16.29 nm^2^, 14.21 nm^2^, 16.12 nm^2^, 14.42 nm^2^, 14.99 nm^2^, 12.92 nm^2^, 16.27 nm^2^, 15.35 nm^2^, 16.87 nm^2^, 17.48 nm^2^ and 12.29 nm^2^ for V5A, D46N, F90V, P103L, R115W, P119L, L215F, P268S, R283Q, R286C, L329F and G331E variants respectively. It is evident from the trace values that while H78Y, R279Q and S340F showed an almost similar trend with each other, the V5A, D46N, F90V, P103L, R115W, P119L, L215F, P268S, R283Q, R286C, L329F and G331E variants behaved differently and exhibited reduced flexibility during the simulations. It was also observed that the majority of the dynamics occurring in these molecular systems was contributed by a small number of eigenvectors representing the overall collective motions. Next, the Gibbs free energy landscape (FEL) plot was generated from the PC1 and PC2 coordinates, where the blue, green, and cyan color represented metastable conformations with a low-energy state, while red color signified high-energy protein conformation. In the FEL plots, the ∆G value of the rare DAAO variants ranged from 13.6 to 16.3 kJ/mol (Fig. 5). This further demonstrated that V5A, D46N, F90V, P103L, R115W, P119L, L215F, P268S, R283Q, R286C, L329F and G331E variants undergone different dynamics as compared to H78Y, R279Q and S340F (Fig. 5).

### RIN of rare DAAO variants

RIN analysis is valuable to identify key residues of interactions as well as perturbed interactions for an individual protein and among a group of proteins during a dynamic run. In this study, we first determined residue-residue interactions between the mutated residues with active site residues and secondly, between the active site loop (residues 216–228) with the rest of the proteins for WT and all the DAAO variants.

Our analysis on residue-residue interactions between variant residues and the active site residues during 100 ns simulations revealed that WT-DAAO possessed several interactions among the active site residues (Figure S7), whereas in D46N variant had fewer interactions in total as a result of the substitution from Asp46 to Asn46 (Figure S7). On the other hand, S340F variant showed relatively higher interactions than the D46N variant (Figure S7). The RINs between the variant and active site residues for the remaining variants have been presented in Figure S8. The differences in residue-residue interactions for the rare variants in relation to the active site residues are summarized in Table S1.

Next, our analysis on the RINs between active site loop residues 216–228 revealed that WT-DAAO has a greater number of interactions than rare variants V5A, D46N, F90V, P103L, R115W, P119L, L215F, P268S, R283Q, R286C, L329F and G331E (Figure S9). It was observed that while WT-DAAO possessed several key interactions such as hydrogen bond between the Arg221‐Asp255, Arg221-Asp127, Glu220-His99, and Asp218-His99 residues, the D46N variant failed to establish these interactions (Figure S9). However, the S340F formed a few unique interactions than the D46N variant showing its ability to exhibit a flexible active site loop (Figure S9). The RINs between the active site loop with the rest of the proteins for the remaining variants are presented in Figure S10.

### Binding free energy calculations and interactions with cofactor FAD

To understand the affinity between rare DAAO variants with cofactor FAD, binding free energies were obtained from respective trajectories of DAAO variants using MM-PBSA method. As shown in Table 3, while the binding free energy of WT was found to be the lowest, followed by H78Y, S340F and R279Q variants, the binding free energies with cofactor FAD for V5A, D46N, F90V, P103L, R115W, P119L, L215F, P268S, R283Q, R286C, L329F and G331E variants were found to be negatively influenced by such variations. A detailed inspection of energy components revealed that the van der Waal and electrostatic energies typically determine the stronger versus the weaker binding affinities towards cofactor FAD. Ligand interaction diagrams of rare variants with cofactor FAD from the average structures further show that L215F and D46N variants established fewer molecular interactions as compared to S340F and R279Q (Figure S11). It was observed that while FAD established ~ 16 types of molecular interactions with L215F and D46N, in S340F and R279Q variants, the FAD established ~ 20 types of molecular interactions with the surrounding residues (Figure S11). This confirms that due to the formation of certain key interactions, variants such as H78Y, R279Q and S340F have higher affinity and favourable binding towards cofactor FAD than the others. Moreover, studies have previously shown that the DAAO apoprotein exists in two conformations at equilibrium in solution, where the state having higher affinity with FAD is significantly enhanced in the presence of ligands, such as benzoate in the active site^21^.

**Table 3:** Comparison of the binding free energy and various energy components between FAD and DAAO variants from respective MD trajectories.

DAAO variants	Van der Waal energy (kJ/mol)	Electrostatic energy (kJ/mol)	Polar solvation energy (kJ/mol)	SASA energy (kJ/mol)	Binding energy (kJ/mol)
WT	− 447.40 ± 24.00	− 353.21 ± 6.17	249.52 ± 8.76	− 33.46 ± 1.29	− 584.55 ± 16.69
V5A	− 444.32 ± 17.67	− 232.44 ± 60.30	232.65 ± 7.27	− 34.53 ± 0.79	− 478.65 ± 39.91
D46N	− 436.01 ± 21.72	− 336.79 ± 22.18	251.93 ± 9.50	− 33.64 ± 1.34	− 554.51 ± 14.90
H78Y	− 445.41 ± 26.11	− 334.65 ± 22.19	243.75 ± 13.51	− 34.23 ± 0.48	− 570.54 ± 33.71
F90V	− 448.14 ± 17.10	− 316.63 ± 28.25	260.21 ± 16.80	− 33.93 ± 0.99	− 538.49 ± 32.27
P103L	− 426.12 ± 9.68	− 310.73 ± 25.50	229.90 ± 8.56	− 34.15 ± 0.57	− 541.10 ± 26.45
R115W	− 444.09 ± 22.70	− 345.75 ± 12.93	265.77 ± 10.52	− 33.57 ± 1.25	− 557.65 ± 11.48
P119L	− 430.99 ± 37.08	− 347.91 ± 13.53	249.92 ± 8.38	− 33.60 ± 1.46	− 562.58 ± 38.96
L215F	− 406.51 ± 16.28	− 344.24 ± 15.30	244.11 ± 2.02	− 33.54 ± 1.78	− 540.18 ± 27.77
P268S	− 436.05 ± 32.92	− 323.31 ± 21.44	244.51 ± 2.99	− 34.48 ± 0.64	− 549.34 ± 29.17
R279Q	− 459.89 ± 6.01	− 314.34 ± 22.80	244.26 ± 3.20	− 33.87 ± 0.36	− 563.84 ± 14.15
R283Q	− 439.31 ± 16.14	− 322.46 ± 24.37	253.11 ± 5.54	− 32.24 ± 0.28	− 540.90 ± 24.59
R286C	− 437.41 ± 28.00	− 320.89 ± 24.64	241.97 ± 10.09	− 32.79 ± 0.81	− 549.12 ± 30.53
L329F	− 457.12 ± 15.21	− 270.67 ± 12.65	227.71 ± 0.67	− 33.79 ± 0.88	− 533.89 ± 17.37
G331E	− 443.19 ± 37.18	− 274.63 ± 8.79	223.01 ± 5.02	− 32.82 ± 0.28	− 527.63 ± 23.14
S340F	− 481.14 ± 8.23	− 279.55 ± 9.72	225.34 ± 0.74	− 32.28 ± 0.23	− 567.64 ± 14.74

### Comparison with experimentally characterized ALS associated DAAO variants

In one of our recent studies, we have effectively shown that certain ALS causing DAAO variants, such as R199W and R199Q, lose their enzymatic activity due to reduced active site loop plasticity and decreased affinity towards FAD^23^. These results highlighted the molecular mechanisms by which the ALS associated DAAO variant, R199W, loses its enzymatic activity and leads to the build-up of D-serine in the ALS spinal cord^14^. It was further observed that R199W and R199Q variants fail to form certain vital salt bridges, such as Glu220-Arg265, Glu100-His217, Glu261-Arg221, Asp255-Arg221 and Asp127-Arg221, which caused the reduced active site loop flexibility, possibly leading to the loss of enzymatic activity. These correlation in structural and dynamic attributes encouraged us to further investigate the Project MinE documented uncharacterized variants of DAAO. Our comprehensive analyses of all the rare DAAO variants showed that V5A, D46N, F90V, P103L, R115W, P119L, L215F, P268S, R283Q, R286C, L329F and G331E rare DAAO variants would lose their enzymatic activity due to an assimilated interplay between restricted active site loop flexibility, distorted interactions with active site residues and eventually due to weak binding towards FAD.

### Limitations of the study

We acknowledge certain limitations in the current study. Our work provides mechanistic insights into the loss-of-function mechanisms for existing mutations using a spectrum of in silico analyses, and hence, comparison with functional assays would be conclusive to support and validate the computational predictions. Moreover, owing to the large number of DAAO variants being discovered frequently, their detailed structure–function analyses through extensive sampling or replica-exchange or longer time-scale simulations would provide a conclusive role of functionally important regions of human DAAO in relation to human diseases.

## Conclusion

Disease causing potential of rare genetic variations cannot be ruled out because of their increased prevalence and susceptibility among ethnic groups, especially in the pathophysiology of complex disorders like ALS. Even if certain rare variants could be tolerated, they may be highly lethal when combined with other variants by exerting a synergistic negative effect on disease development. One such emerging protein associated with ALS is DAAO, where the number of rare variants is enormous, though their structure–function relationship and possible role in loss-of-functions have never been established. We have earlier shown that missense mutations in DAAO cause ALS through loss-of-function mechanisms and during the course of this research, we realized there are several ALS associated rare variants of DAAO for which the mechanisms of disease association are not understood. Rare variants act as risk factors and disease modifiers to increase disease burden and thus investigating their role is of great importance to define the genetic architecture of ALS. Further, predicting whether a novel variant is deleterious or benign, is one of essential tasks with respect to disease pathophysiology. We were curious to comprehend if certain structural and dynamic attributes can be identified from our comprehensive in silico analyses, which can be used to distinguish a deleterious/benign variant and provide insights into their functional loss mechanisms and subsequently ALS causation.

Although a multidisciplinary approach is the ultimate solution to this complexity, extensive in-silico analyses-based approaches will be of immense use in shortlisting and prioritizing deleterious variants for further clinical and biological studies. To fill this gap, we mined all the rare DAAO variants documented in ProjectMinE. Through extensive analyses, we found that some of the variants should have a collective consequence on the active site loop, and cofactor binding of DAAO, all of which play a key role in negatively affecting the enzymatic function. This report along with previous works by us and other researchers demand that whole exome sequencing of ALS patients across diverse geographical regions is required to identify novel DAAO variants in order to develop effective therapeutic strategies. This work gives us a set of structural and dynamic attributes, which we can quickly compute and evaluate for any novel DAAO variant and predict their loss-of-function mechanisms. Our computational methodology can be expanded to other ALS relevant protein families and neurodegenerative disorders to bridge the gap between variant identification, characterization and disease manifestation through a better insight into their structure-dynamics-function relationships.

## Methods

### Retrieval of rare variants of DAAO

Rare DAAO variants were retrieved from the Project MinE data browser and then filtered for our analysis as follows. First, only the missense variants with rare variant burden of 0.5% were extracted and variants for which the functional mechanisms are not known were selected for our study. Out of a total of 20 variants (V5A, R38H, D46N, H78Y, F90V, P103L, R115W, P119L, R199W, R199Q, L215F, P268S, R279Q, R283Q, R286C, L329F, G331E, S340F, S345F and S345C), the R38H, R199W and R199Q variants were not included in this study because they were previously studied by us and others^13,14,23^. Further, variants S345F and S345C were excluded because the crystal structure of WT-DAAO (PDB ID: 2E49) contains residues until Ser340 only. The allele frequency data retrieved for these variants indicated that they qualify as rare variants (MAF < 0.05%). Next, the human DAAO (Accession number: P14920) protein sequence was retrieved from UniProtKB to locate the position of source residues, which were then altered to the mutated residues.

### Modeling of rare DAAO variants and all-atom MD simulations

The co-crystal structure of WT-DAAO bound to FAD and imino-serine (PDB ID: 2E49) was used as the starting structure for all-atom MD simulations^24,25^. As the crystal structures of the rare variants of DAAO were not available, the variant structures were modelled using the *swapaa* tool of UCSF Chimera and the models were evaluated using PROCHECK for stereochemical quality assessment^26,27^. In total, 15 rare variants of DAAO including V5A, D46N, H78Y, F90V, P103L, R115W, P119L, L215F, P268S, R279Q, R283Q, R286C, L329F, G331E and S340F were modelled and considered in this study. First, the topologies for FAD and imino-serine were assigned using ATB Repository and the resulting complexes were added with hydrogen atoms^28,29^. Each molecular system was then solvated in a dodecahedron box of simple point charge (SPC) water in the center at a minimum distance of 1.0 nm between the protein surface and box boundary^30^. Following solvation, each system was electrostatically neutralized and simulations were carried out with gromos96 54A7 force field using GROMACS 5.1.2^31–33^. Each simulation was performed by employing 50,000 steps of energy minimization followed by a gradual heating from 0 to 300 K in 200 ps and constant temperature equilibration for 1000 ps at 300 K. During this, the Parrinello-Rahman barostat pressure coupling was used to avoid the impact of velocity^34^. Upon the completion of these steps, each system was inspected for equilibration at the desired temperature and pressure, and subsequently, 100 ns production runs were carried out for each rare variant with a periodic boundary condition in the NPT ensemble with modified Berendsen temperature coupling at 300 K and a constant pressure of 1 atm^35^. The leap-frog integrator and the Verlet cut-off schemes were employed. The LINCS algorithm was used to constrain the bond lengths and the Particle-mesh Ewald method was employed to calculate the electrostatic forces^36,37^.

### Analysis of MD simulations

The MD simulated trajectories of rare DAAO variants were analyzed using gmx rms, gmx rmsf and gmx gyrate GROMACS utilities to obtain the root mean square deviation (RMSD), root mean square fluctuation (RMSF), and radius of gyration (Rg) respectively. The intermolecular hydrogen bonds and salt bridges were computed using visual molecular dynamics (VMD)’s hydrogen bonds and salt bridge modules respectively. The formation or dissolution of salt bridges with respect to time was processed using the “timeline” plugin of VMD^38^. The secondary structure content for the rare DAAO variants during the course of the simulations were measured using the DSSP (Dictionary of Secondary Structure of Proteins) tool of the GROMACS 'do_dssp' utility.

### Essential dynamics and free energy landscape of DAAO rare variants

To understand the prevailing and cooperative modes of the rare DAAO variants from the complete dynamics of the MD trajectories, principal component analysis (PCA) was performed, where a variance/covariance matrix was constructed by calculating the eigenvectors and eigenvalues and their projection along the first two principal components^39^. Briefly, PCA is based on the diagonalization of the covariance matrix *C*, with the elements defined as follows:

$$C_{{ij}} \le (r_{i} - < r_{i} > )*(r_{j} - < r_{j} {\text{ > }})\,\,\,(i,j = 1,2,3, \ldots 3N) $$

where *r*_*i*_ is the Cartesian coordinate of the *i*th C_⍺_ atom, *N* is the number of C_⍺_ atoms considered, and < r_*i*_ > represents the time average over all the configurations obtained in the simulation. By projecting the MD trajectories of the variants onto the main essential direction, which corresponds to the largest eigenvector, one can visualize the extreme structures and the major fluctuations of the correlated motions. Following this, the movements of the variants in the essential subspace were identified by the projection of Cartesian coordinates across the trajectories. The percentage variability of the rare DAAO variants was calculated by the eigenvalues associated with each eigenvector. The eigenvalues and eigenvectors were obtained by diagonalizing the covariance matrix. Further, the conformational changes of the rare variants free energy landscape (FEL) were computed using gmx sham package^40,41^.

### Residue interaction network (RIN) of DAAO rare variants

To understand the effect of variations on overall interacting residues of the rare DAAO variants, RINs were constructed using an integrated computational framework comprising UCSF Chimera, structureViz and RINalyzer plugins of Cytoscape^42–45^. For this, UCSF Chimera was used to load the MD trajectories of the rare variants and its RIN wizard was used to compute the residue interactions and then visualized using structureViz. In the network diagrams, the amino acids were represented by nodes and non-covalent interactions by edges. Furthermore, contacts, hydrogen bonds, backbone connectivity and distances were retrieved from each rare variant. After the RINs were generated, they were visualized by the “yFiles Tree layout” in Cytoscape. For each DAAO variant, two interaction networks were generated.

### Binding free energy between rare DAAO variants and FAD

For calculating the binding free energies between rare DAAO variants and FAD, molecular mechanics/Poisson Boltzmann surface area (MM-PBSA) methodology embedded in the g_mmpbsa tool of GROMACS was used^46,47^. In MM-PBSA, the binding free energy of the protein and ligand is typically defined as

$$ \Delta G_{{\rm binding}} = \Delta G_{{\rm complex}} - (\Delta G_{{\rm protein}}+\Delta G_{{\rm ligand}})$$

where ΔG_complex_, ΔG_protein_, and ΔG_ligand_ represent the total free energies of the protein–ligand complex, the protein and the ligand separately in the solvent respectively^48,49^. Moreover, the free energy of the individual entity is symbolized as

$$G = E_{{\rm MM}} + G_{{\rm solvation}} -{\text{TS}}$$

where E_MM,_ G_solvation_ and TS represent the average molecular mechanics potential energy in the vacuum, free energy of solvation, and entropic contribution to the free energy in vacuum respectively. The T and S denotes the temperature and entropy, respectively. Further, the E_MM_ consists of bonded and nonbonded terms including bond angle, torsion, and electrostatic (E_elec_) and the VDW (E_vdw_) interactions respectively. Lastly, the solvation free energy, G_solvation_ considers both electrostatic and non-electrostatic (G_polar_ and G_nonpolar_) components. The binding free energies of each system with FAD were calculated for 400 snapshots taken from the final 30 ns of the simulation.

### Figures and rendering

Relevant figures were rendered and generated using PyMOL and VMD. Cytoscape was used for the generation of RINs, and ligand interaction diagrams were generated using Molecular Operating Environment (MOE) software version 2018.01.

## Supplementary information

Supplementary Information 1.
