# Supplementary material for: Mechanistic insights into the loss-of-function mechanisms of rare human D-amino acid oxidase variants implicated in amyotrophic lateral sclerosis
Source: Sci Rep. 2020 Oct 13;10:17146. doi: 10.1038/s41598-020-74048-2 (PMC7555490; doi:10.1038/s41598-020-74048-2)
Supplement: Supplementary file 1 — Supplementary Information 1. [file 41598_2020_74048_MOESM1_ESM.pdf]

SUPPORTING INFORMATION

Mechanistic insights into the loss-of-function mechanisms of rare human D-amino acid oxidase variants implicated in amyotrophic lateral sclerosis

Aditya K. Padhi[1], Kam Y. J. Zhang[1*]

[1]Laboratory for Structural Bioinformatics, Center for Biosystems Dynamics Research, RIKEN, 1-7-22 Suehiro, Tsurumi, Yokohama, Kanagawa, 230-0045, Japan

*Corresponding author:

Kam Y. J. Zhang

Email: kamzhang@riken.jp

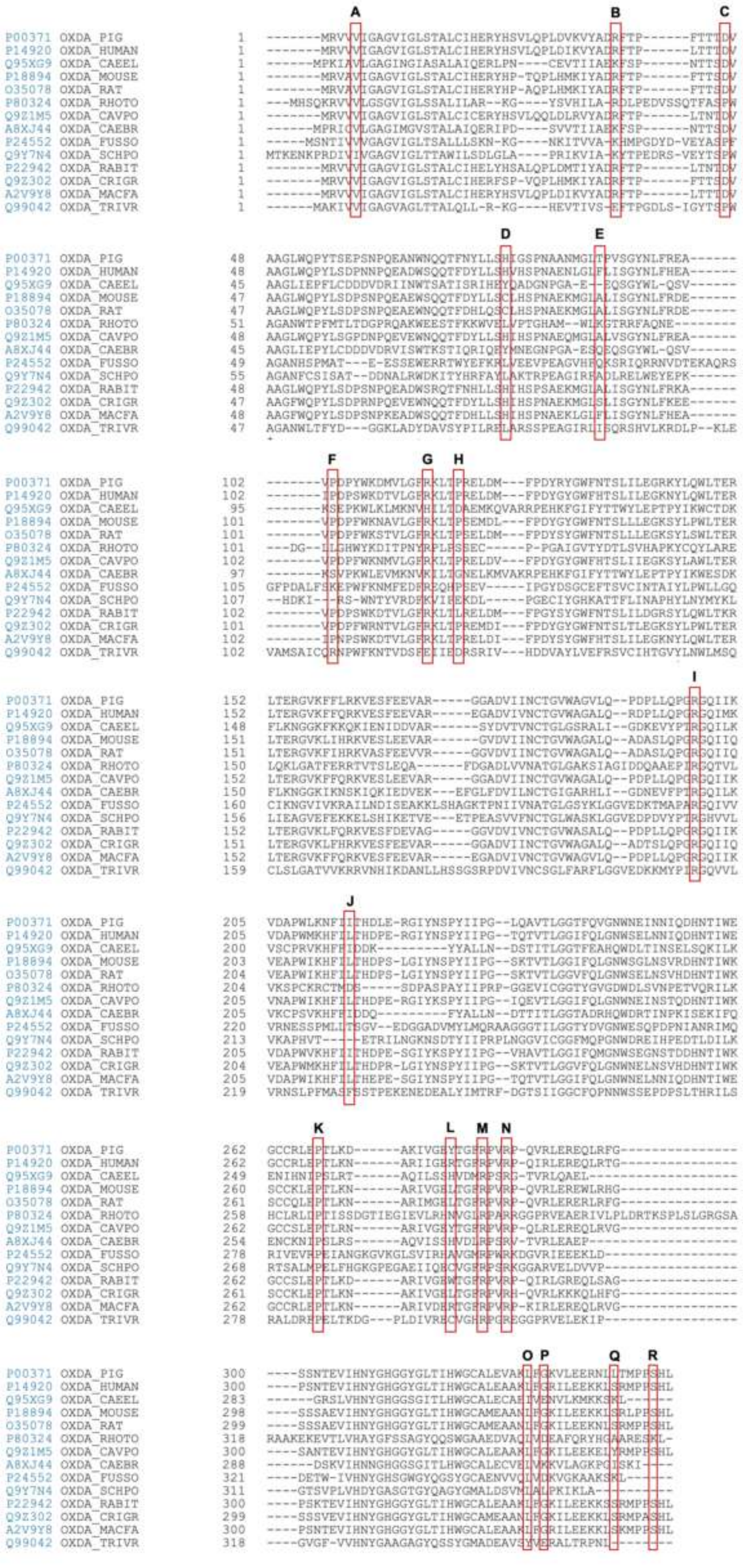

Figure S1. Conservation of amino acid residues in DAAO across 14 species. The positions of Val5, Arg38, Asp46, His78, Phe90, Pro103, Arg115, Pro119, Arg199, Leu215, Pro268, Arg279, Arg283, Arg286, Leu329, Gly331, Ser340 and Ser345 are shown in red boxes, along with their Uniprot accession numbers, ranged from (A-R) respectively.

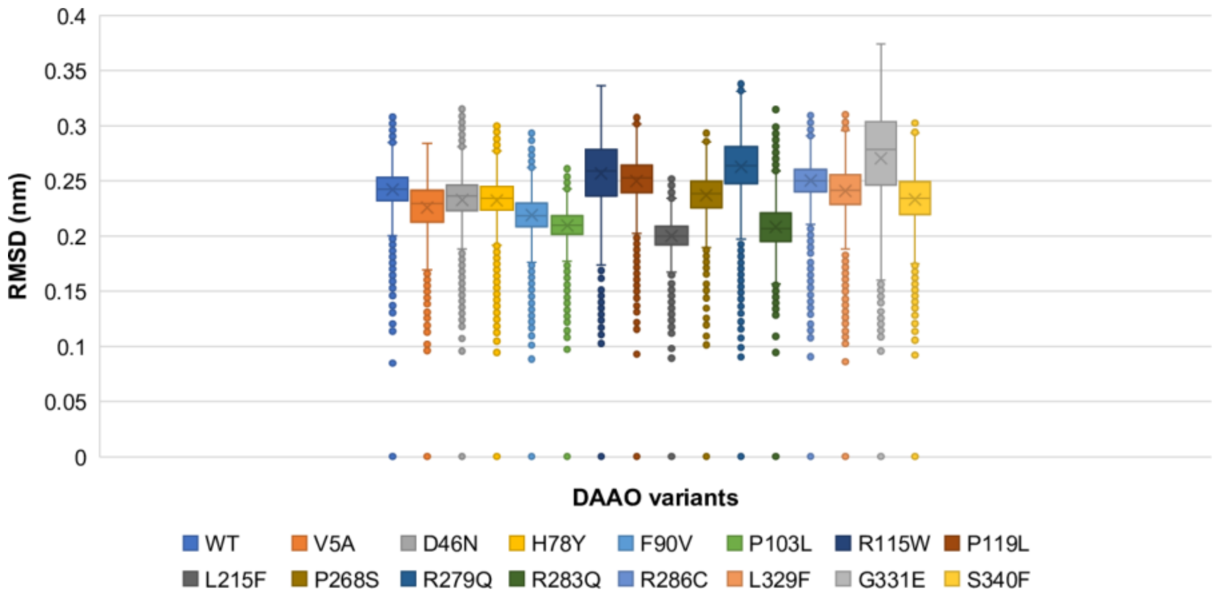

Figure S2. Boxplots showing the backbone RMSD profiles of WT and rare DAAO variants during the course of MD simulations**.**

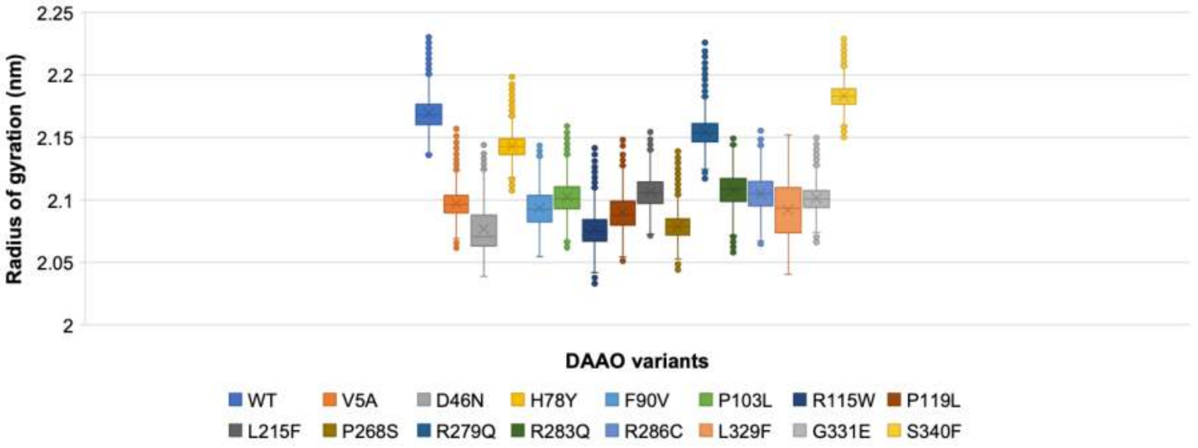

Figure S3. Boxplots showing the Radius of gyration of Cα atoms for WT and DAAO variants as a function of time.

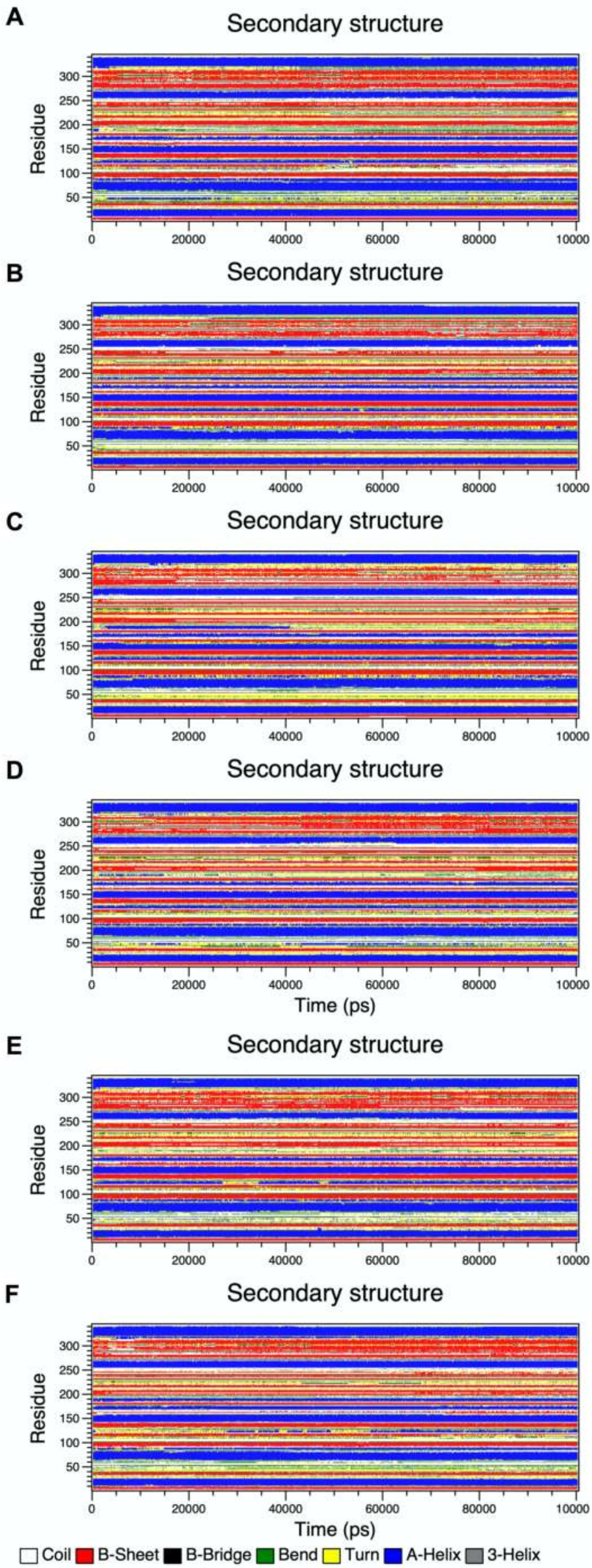

A
Secondary structure
Residue
300
250
200
150
100
50
0
20000
40000
60000
80000
10000
B
Secondary structure
C
Secondary structure
D
Secondary structure
Time (ps)
E
Secondary structure
F
Secondary structure
Time (ps)
Coil
B-Sheet
B-Bridge
Bend
Turn
A-Helix
3-Helix

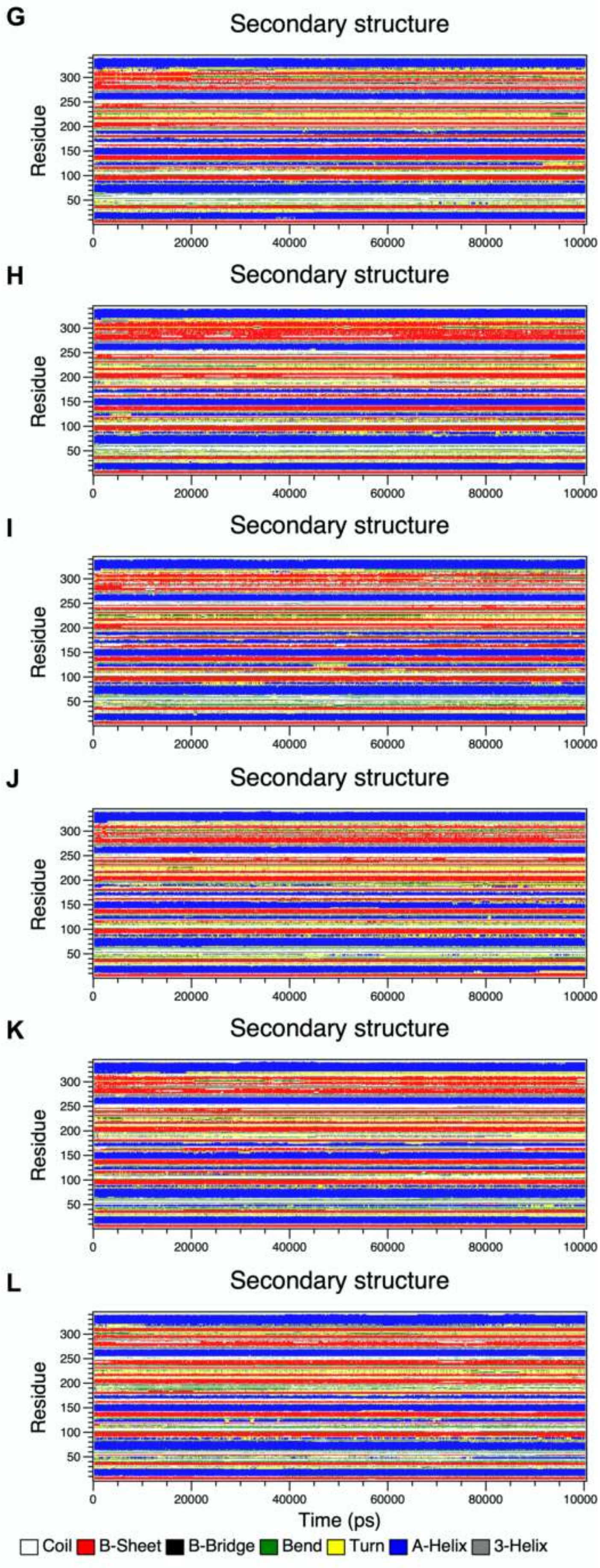
G
Secondary structure
H
Secondary structure
I
Secondary structure
J
Secondary structure
K
Secondary structure
L
Secondary structure
Residue
300
250
200
150
100
50
0
20000
40000
60000
80000
10000
Time (ps)
Coil
B-Sheet
B-Bridge
Bend
Turn
A-Helix
3-Helix

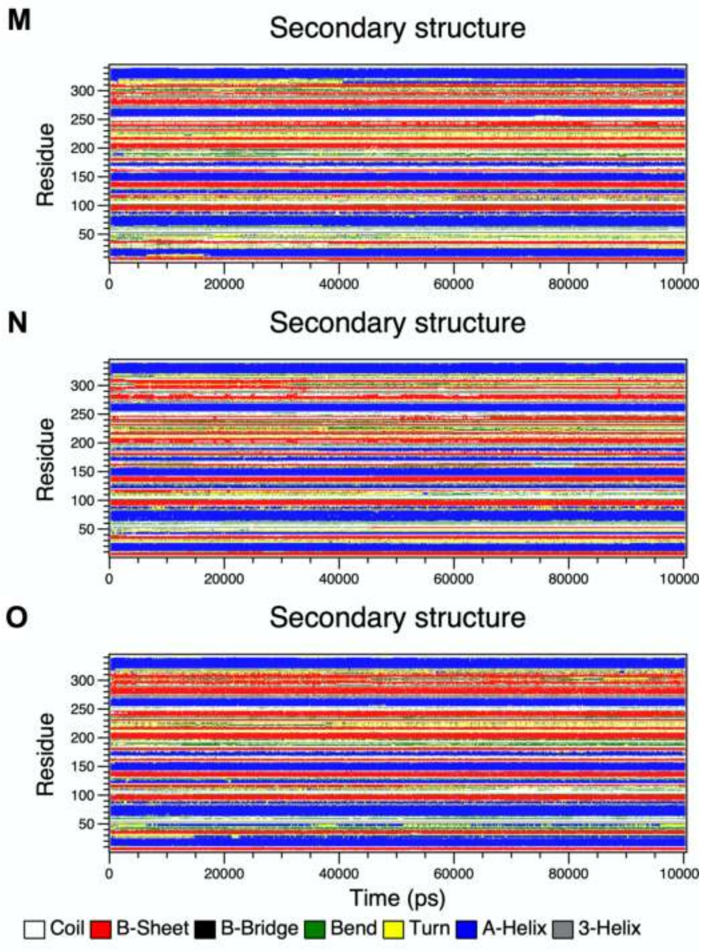

Figure S4. Secondary structure of the rare DAAO variants. Secondary structure elements of the rare DAAO variants as a function of time during MD simulations. V5A, D46N, H78Y, F90V, P103L, R115W, P119L, L215F, P268S, R279Q, R283Q, R286C, L329F, G331E and S340F variants are represented as (A to O).

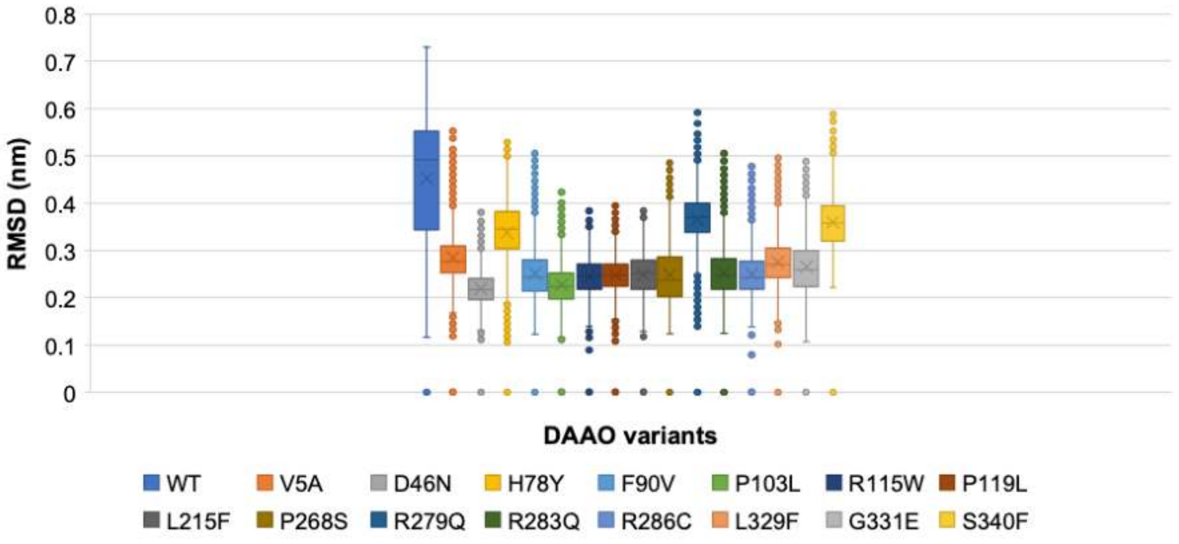

Figure S5. Boxplots showing the time evolution RMSD profiles of WT and DAAO variants for the active site loop are shown.

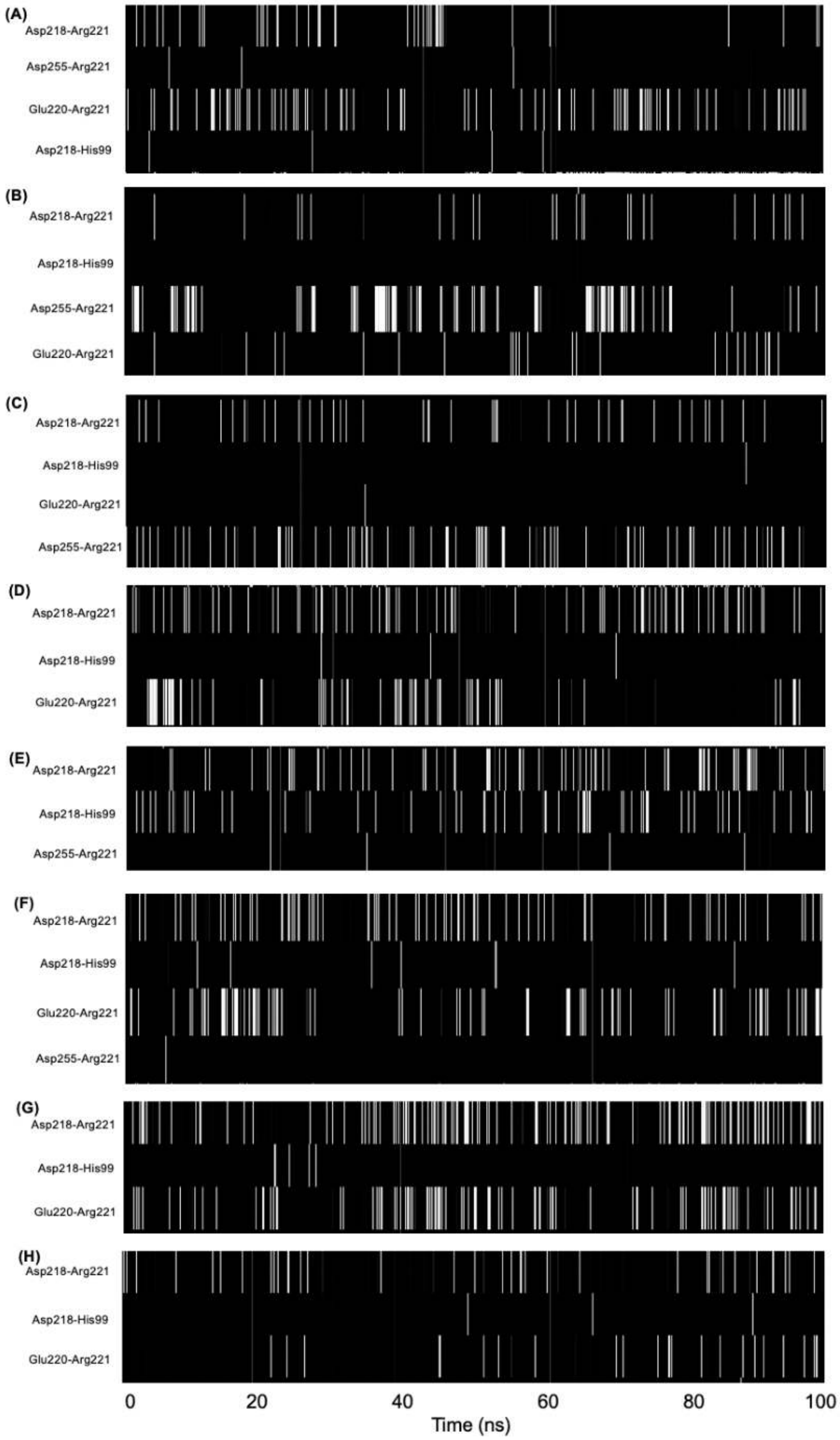
(A)
Asp218-Arg221
Asp255-Arg221
Glu220-Arg221
Asp218-His99
(B)
Asp218-Arg221
Asp218-His99
Asp255-Arg221
Glu220-Arg221
(C)
Asp218-Arg221
Asp218-His99
Glu220-Arg221
Asp255-Arg221
(D)
Asp218-Arg221
Asp218-His99
Glu220-Arg221
(E)
Asp218-Arg221
Asp218-His99
Asp255-Arg221
(F)
Asp218-Arg221
Asp218-His99
Glu220-Arg221
Asp255-Arg221
(G)
Asp218-Arg221
Asp218-His99
Glu220-Arg221
(H)
Asp218-Arg221
Asp218-His99
Glu220-Arg221
0
20
40
60
80
100
Time (ns)

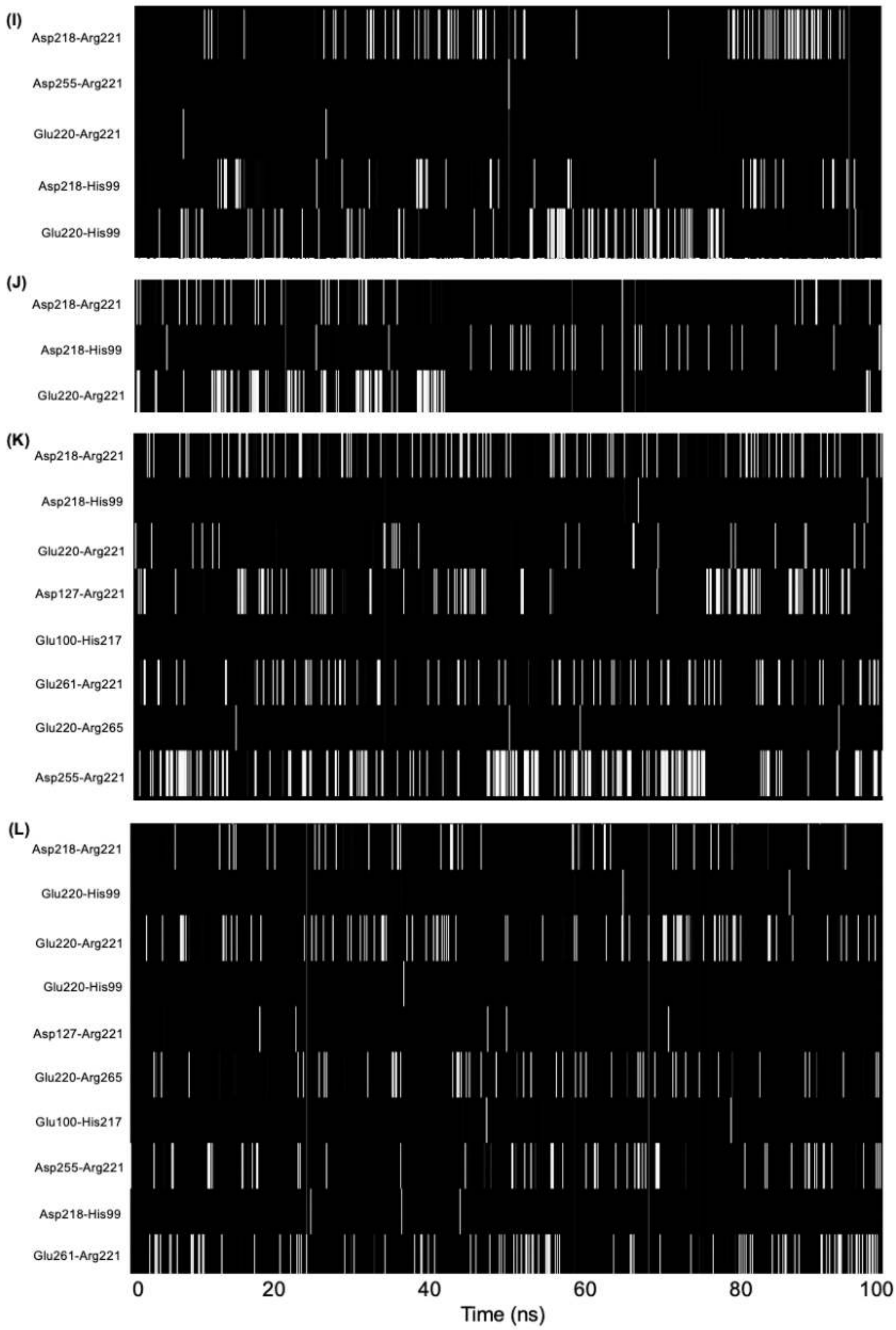

Figure S6. Time evolution of salt bridge interactions of rare DAAO variants. Formation or dissolution of salt bridge interactions of DAAO variants (V5A, F90V, P103L, R115W, P119L, P268S, R283Q, R286C, L329F, G331E, H78Y) during MD simulations is shown from (A-L). White bar indicates the formation of salt bridge during the 100 ns simulations.

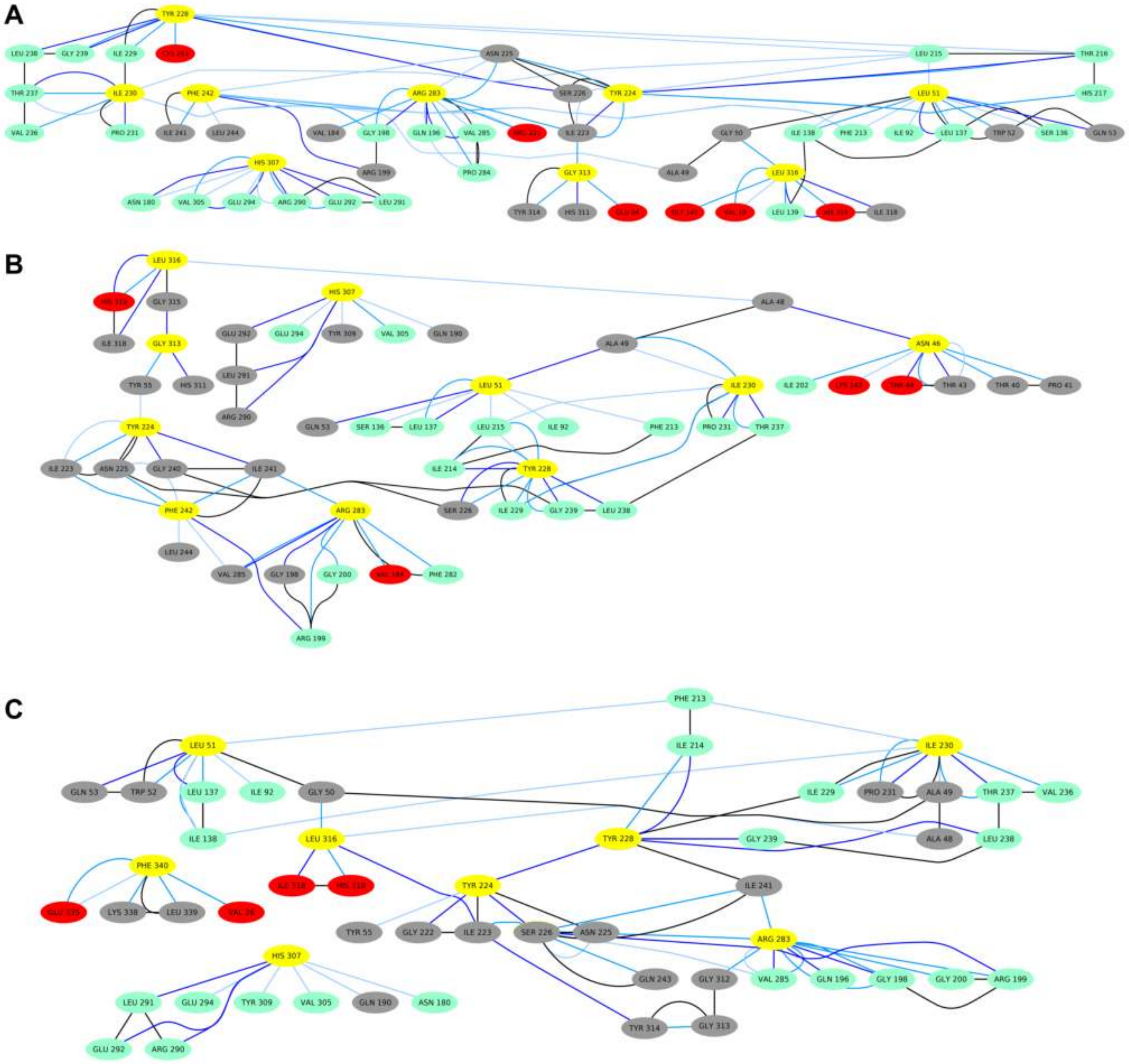

Figure S7. RINs between WT and rare DAAO variants for the active site residues. Comparison of RINs between (A) WT, (B) D46N and (C) S340F highlighting the changes in network interaction for enzymatically important active site residues Leu51, Tyr224, Tyr228, Ile230, Phe242, Arg283, His307, Gly313, and Leu316. Amino acids are represented as nodes and interaction types are represented as edges. Blue color edge denotes the hydrogen-bonds and black color edge denotes the contact between the residues. Mutated and active site residues are shown in yellow colored nodes and the remaining nodes are shown according to their secondary structure type; helix: red, sheet: light green, and loop: gray color.

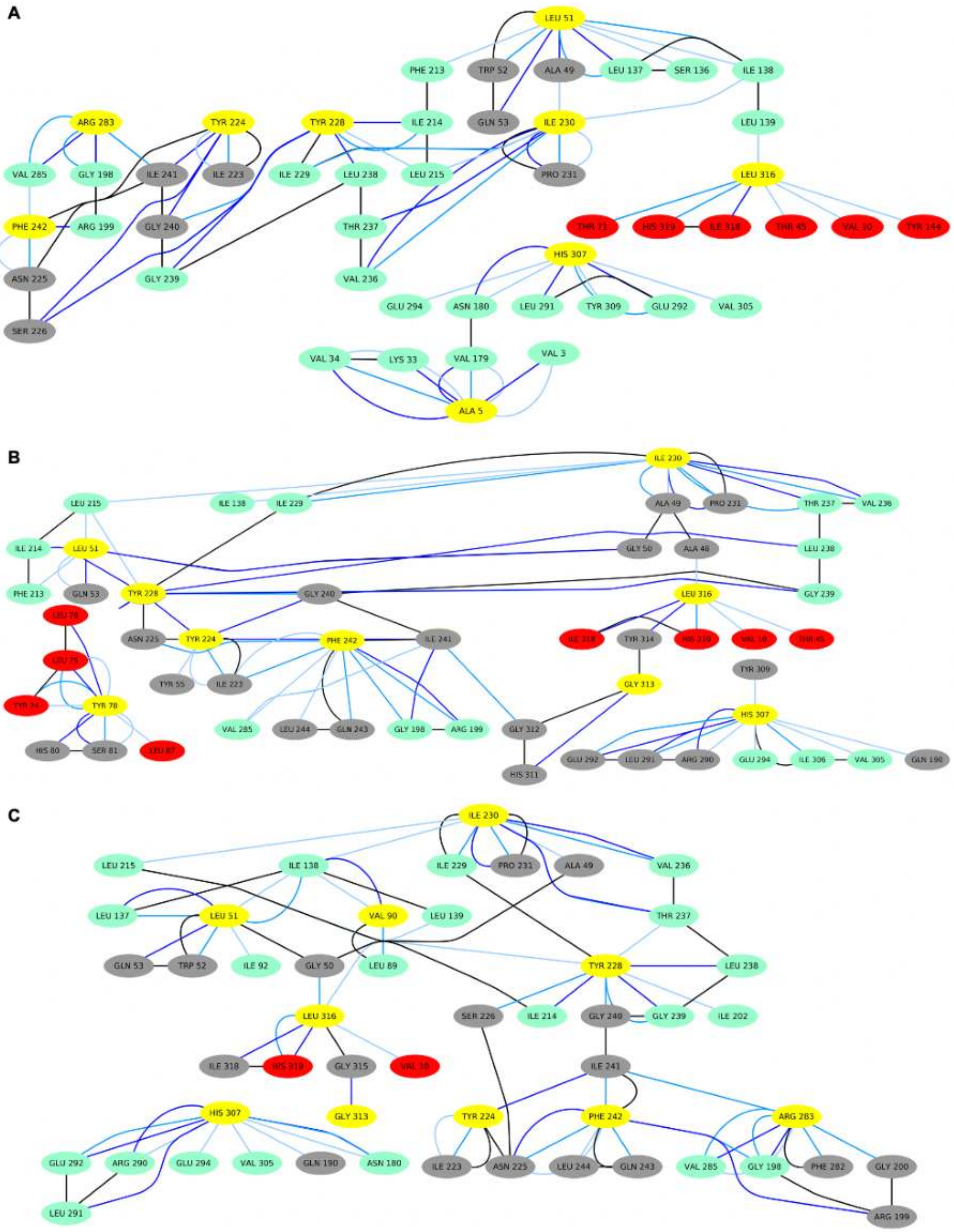
A
LEU 51
PHE 213
TRP 52
ALA 49
LEU 137
SER 136
ILE 138
ARG 283
TYR 224
TYR 228
ILE 214
GLN 53
ILE 230
LEU 139
VAL 285
GLY 198
ILE 241
ILE 223
ILE 229
LEU 238
LEU 215
PRO 231
LEU 316
PHE 242
ARG 199
GLY 240
THR 237
THR 71
HIS 319
ILE 318
THR 45
VAL 10
TYR 144
ASN 225
GLY 239
VAL 236
HIS 307
SER 226
GLU 294
ASN 180
LEU 291
TYR 309
GLU 292
VAL 305
VAL 34
LYS 33
VAL 179
VAL 3
ALA 5
B
ILE 230
LEU 215
ILE 138
ILE 229
ALA 49
PRO 231
THR 237
VAL 236
ILE 214
LEU 51
GLY 50
ALA 48
LEU 238
PHE 213
GLN 53
TYR 228
GLY 240
LEU 316
GLY 239
ASN 225
TYR 224
PHE 242
ILE 241
ILE 318
TYR 314
HIS 319
VAL 10
THR 45
TYR 55
ILE 223
TYR 309
GLY 313
TYR 78
HIS 307
VAL 285
LEU 244
GLN 243
GLY 198
ARG 199
GLY 312
HIS 80
SER 81
GLU 292
LEU 291
ARG 290
GLU 294
ILE 306
VAL 305
GLN 190
HIS 311
C
ILE 230
LEU 215
ILE 138
ILE 229
PRO 231
ALA 49
VAL 236
LEU 137
LEU 51
VAL 90
LEU 139
THR 237
GLN 53
TRP 52
ILE 92
GLY 50
LEU 89
TYR 228
LEU 238
LEU 316
SER 226
ILE 214
GLY 240
GLY 239
ILE 202
ILE 318
HIS 319
GLY 315
VAL 10
ILE 241
HIS 307
GLY 313
TYR 224
PHE 242
ARG 283
GLU 292
ARG 290
GLU 294
VAL 305
GLN 190
ASN 180
ILE 223
ASN 225
LEU 244
GLN 243
VAL 285
GLY 198
PHE 282
GLY 200
LEU 291
ARG 199

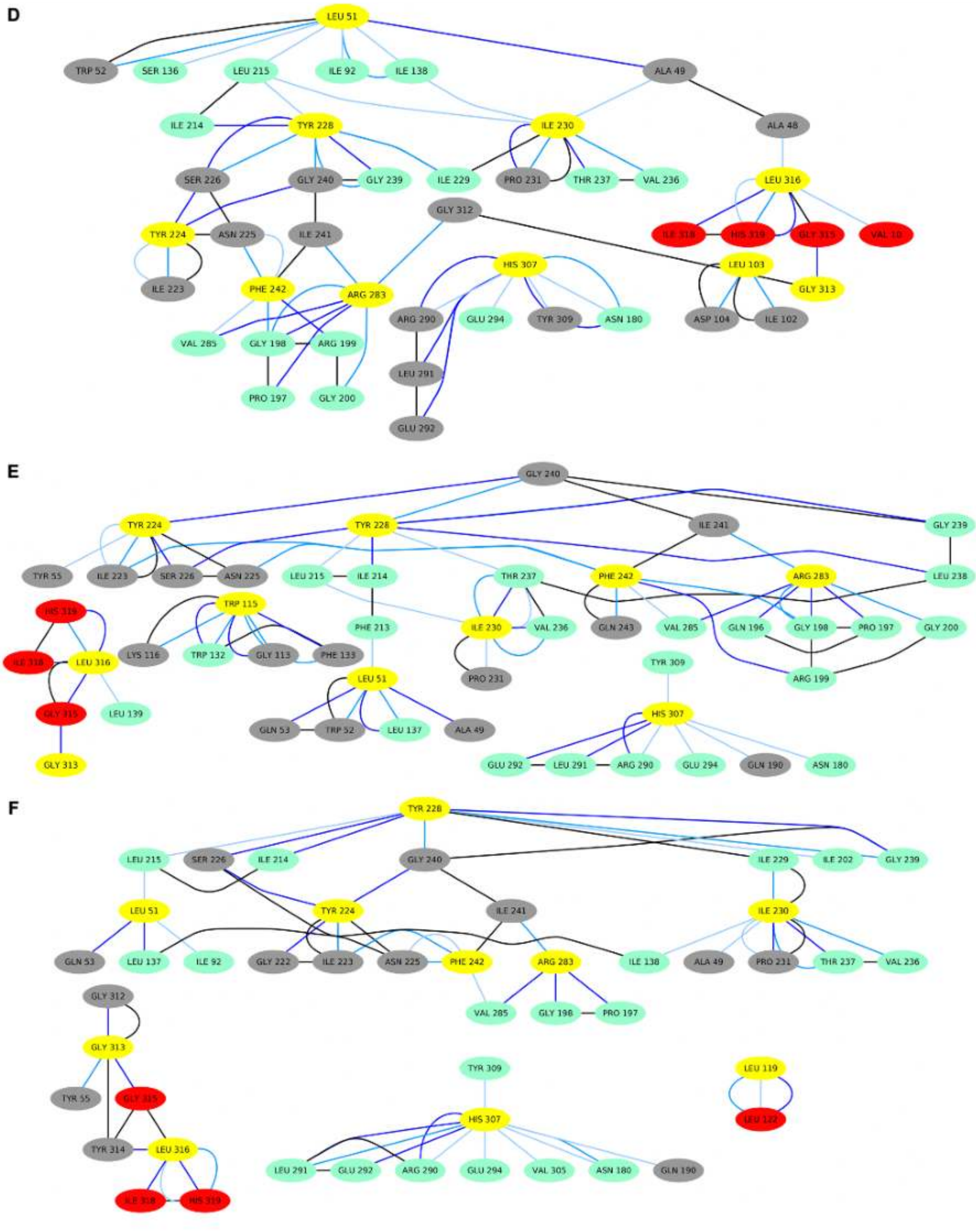
D
LEU 51
TRP 52
SER 136
LEU 215
ILE 92
ILE 138
ALA 49
ILE 214
TYR 228
ILE 230
ALA 48
SER 226
GLY 240
GLY 239
ILE 229
PRO 231
THR 237
VAL 236
LEU 316
GLY 312
TYR 224
ASN 225
ILE 241
ILE 318
HIS 319
GLY 315
VAL 10
HIS 307
LEU 103
ILE 223
PHE 242
ARG 283
GLY 313
ARG 290
GLU 294
TYR 309
ASN 180
ASP 104
ILE 102
VAL 285
GLY 198
ARG 199
LEU 291
PRO 197
GLY 200
GLU 292
E
GLY 240
TYR 224
TYR 228
ILE 241
GLY 239
TYR 55
ILE 223
SER 226
ASN 225
LEU 215
ILE 214
THR 237
PHE 242
ARG 283
LEU 238
HIS 319
TRP 115
ILE 318
LEU 316
LYS 116
TRP 132
GLY 113
PHE 133
PHE 213
ILE 230
VAL 236
GLN 243
VAL 285
GLN 196
GLY 198
PRO 197
GLY 200
PRO 231
TYR 309
ARG 199
LEU 51
GLY 315
LEU 139
GLN 53
TRP 52
LEU 137
ALA 49
HIS 307
GLY 313
GLU 292
LEU 291
ARG 290
GLU 294
GLN 190
ASN 180
F
TYR 228
LEU 215
SER 226
ILE 214
GLY 240
ILE 229
ILE 202
GLY 239
LEU 51
TYR 224
ILE 241
ILE 230
GLN 53
LEU 137
ILE 92
GLY 222
ILE 223
ASN 225
PHE 242
ARG 283
ILE 138
ALA 49
PRO 231
THR 237
VAL 236
GLY 312
VAL 285
GLY 198
PRO 197
GLY 313
TYR 309
LEU 119
TYR 55
GLY 315
HIS 307
LEU 122
TYR 314
LEU 316
LEU 291
GLU 292
ARG 290
GLU 294
VAL 305
ASN 180
GLN 190
ILE 318
HIS 319

G

H

I

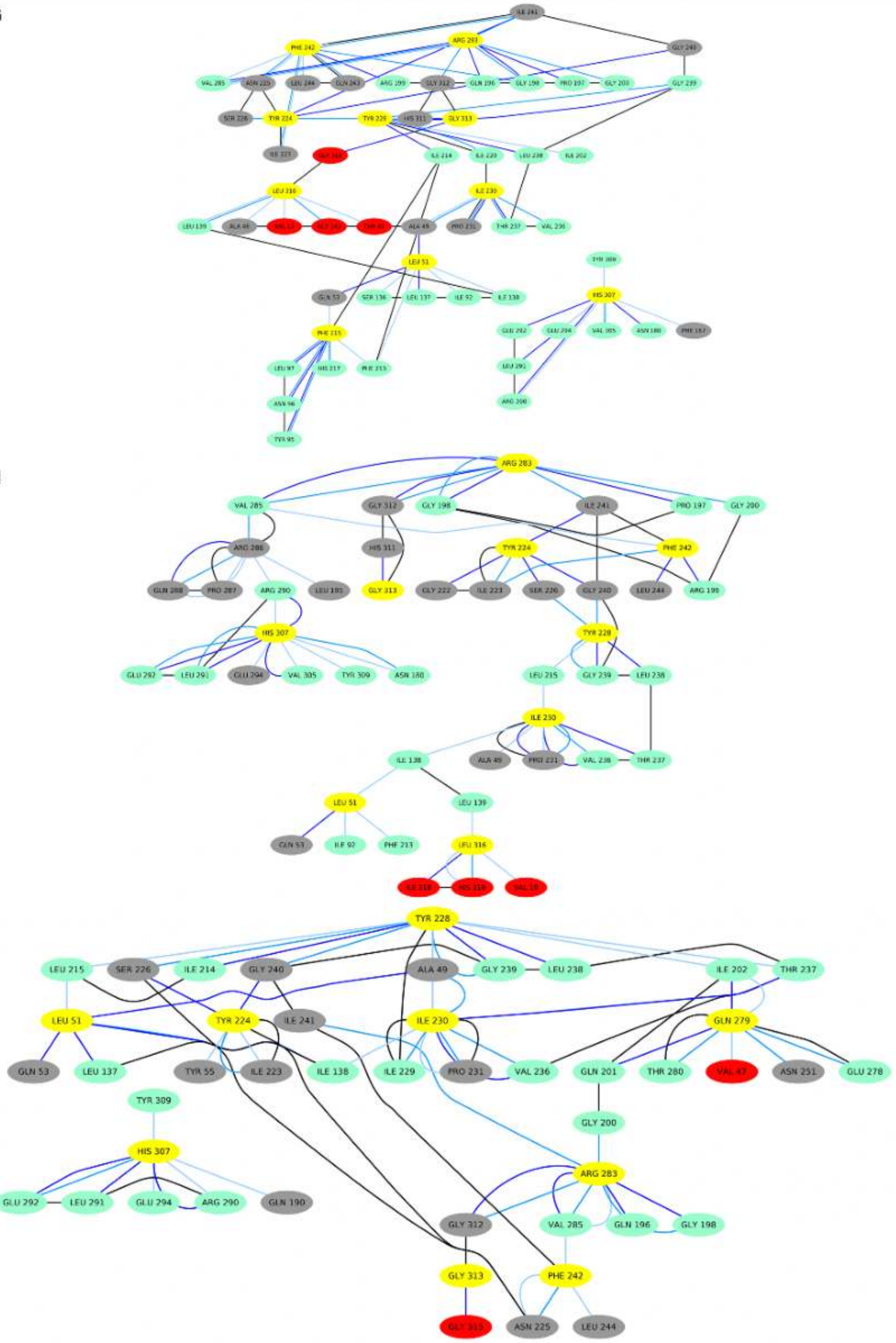
ARG 283
VAL 285
GLY 312
GLY 198
ILE 241
PRO 197
GLY 200
ARG 286
HIS 311
TYR 224
PHE 242
GLN 288
PRO 287
ARG 290
LEU 185
GLY 313
GLY 222
ILE 223
SER 226
GLY 240
LEU 244
ARG 199
HIS 307
TYR 228
GLU 292
LEU 291
GLU 294
VAL 305
TYR 309
ASN 180
LEU 215
GLY 239
LEU 238
ILE 230
ILE 138
ALA 49
PRO 231
VAL 236
THR 237
LEU 51
LEU 139
GLN 53
ILE 92
PHE 213
LEU 316
SER 226
ILE 214
ALA 49
ILE 202
GLN 279
LEU 137
TYR 55
ILE 229
GLN 201
THR 280
ASN 251
GLU 278
GLY 200
GLN 190
GLN 196
ASN 225

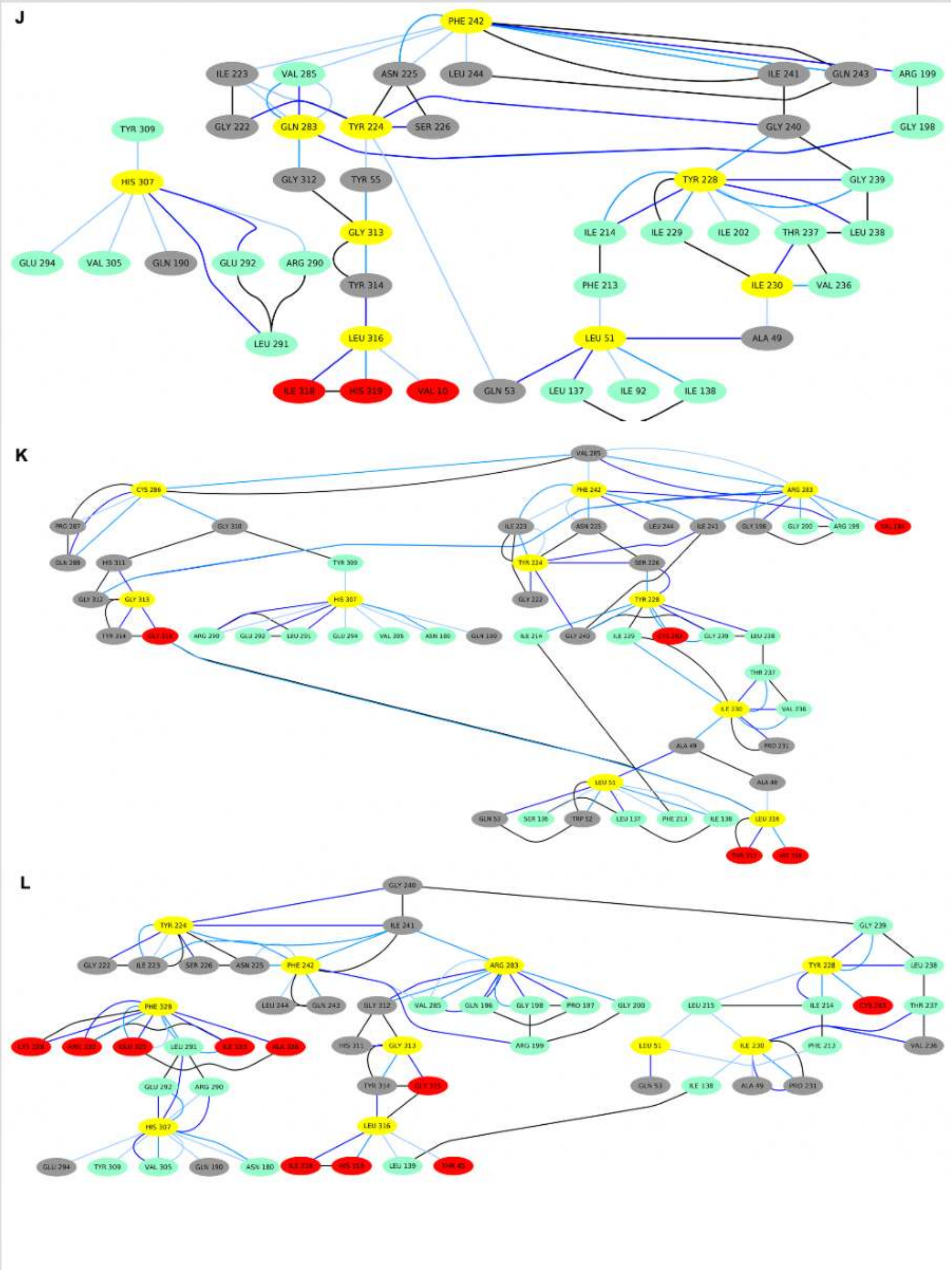
J
PHE 242
ILE 223
VAL 285
ASN 225
LEU 244
ILE 241
GLN 243
ARG 199
TYR 309
GLY 222
GLN 283
TYR 224
SER 226
GLY 240
GLY 198
HIS 307
GLY 312
TYR 55
TYR 228
GLY 239
GLY 313
ILE 214
ILE 229
ILE 202
THR 237
LEU 238
GLU 294
VAL 305
GLN 190
GLU 292
ARG 290
TYR 314
PHE 213
ILE 230
VAL 236
LEU 291
LEU 316
LEU 51
ALA 49
ILE 318
HIS 319
VAL 10
GLN 53
LEU 137
ILE 92
ILE 138
K
L

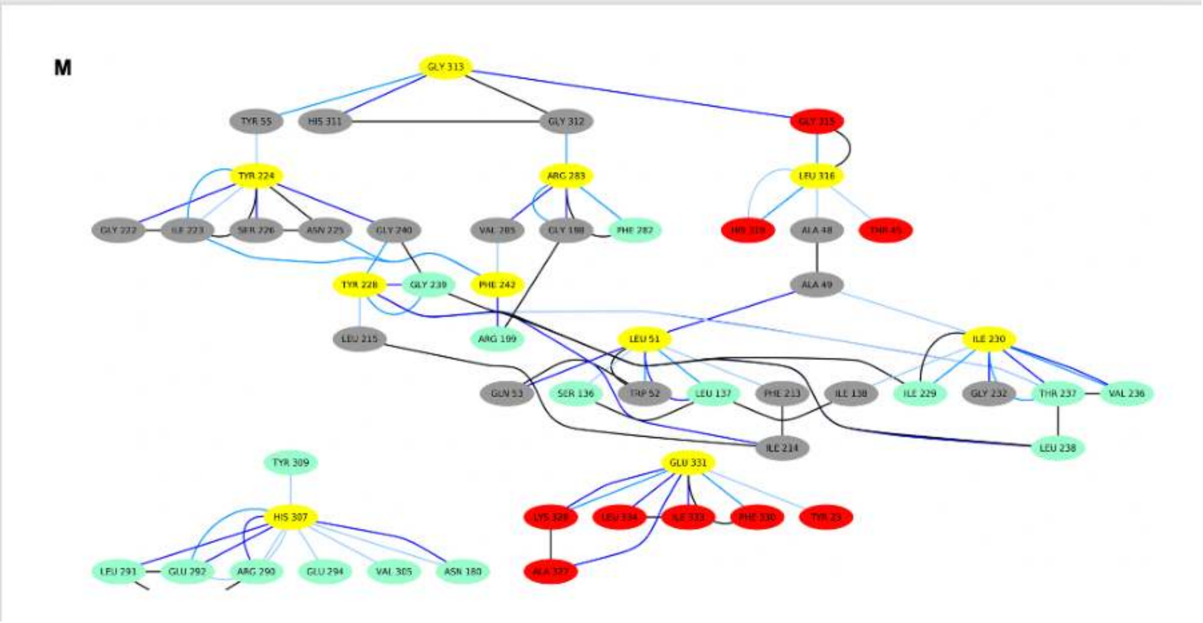

Figure S8. Residue interaction networks between wild-type and rare DAAO variants for the active site residues. Comparison of residue interaction networks between (A-M) for variants (V5A, H78Y, F90V, P103L, R115W, P119L, L215F, P268S, R279Q, R283Q, R286C, L329F, G331E) highlighting the changes in network interaction for enzymatically important active site residues Leu51, Tyr224, Tyr228, Ile230, Phe242, Arg283, His307, Gly313, and Leu316. Amino acids are represented as nodes and interaction types are represented as edges. Blue color edge denotes the hydrogen-bonds and black color edge denotes the contact between the residues. Mutated and active site residues are shown in yellow colored nodes and the remaining nodes are shown according to their secondary structure type; helix: red, sheet: light green, and loop: gray color.

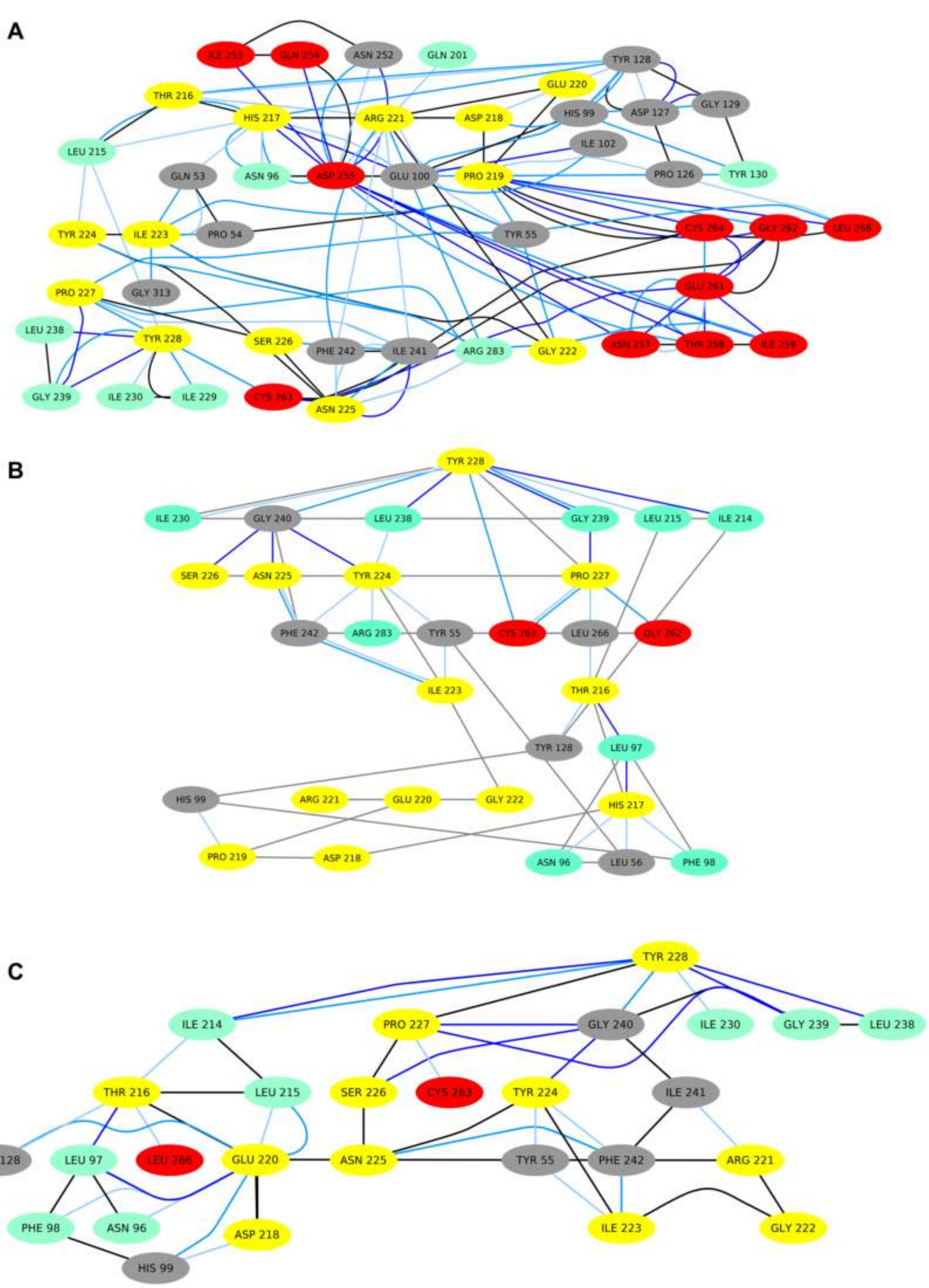

Figure S9. RINs between WT and rare DAAO variants for the active site loop comprising 216-228 residues. Comparison of RINs between (A) WT, (B) D46N and (C) S340F highlighting the changes in network interaction for active site loop comprising 216-228 residues. Amino acids are represented as nodes and interaction types are represented as edges. Blue color edge denotes the hydrogen-bonds and black color edge denotes the contact between the residues. Active site loop residues are shown in yellow colored nodes and the remaining nodes are shown according to their secondary structure type; helix: red, sheet: light green, and loop: gray color.

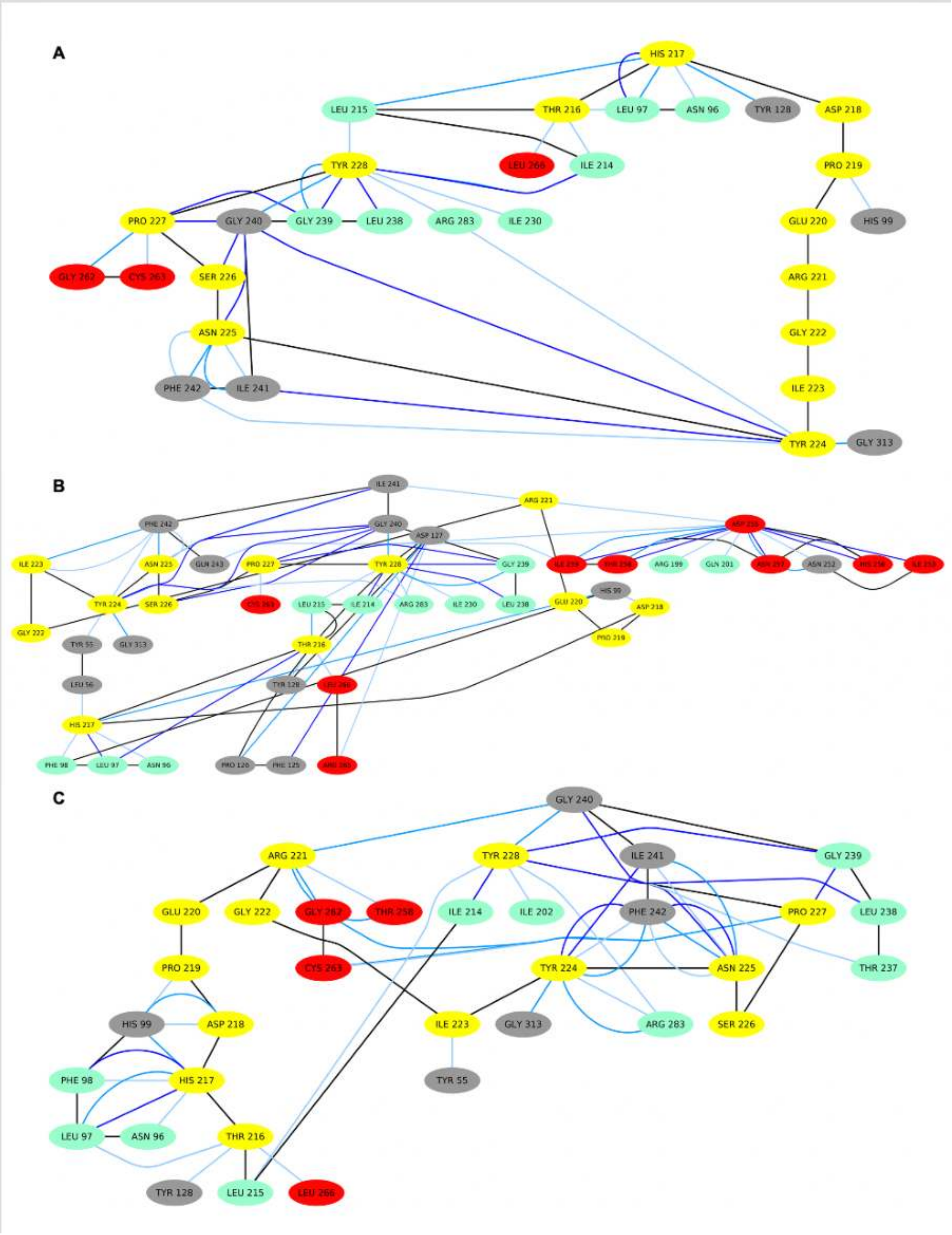
A
HIS 217
LEU 215
THR 216
LEU 97
ASN 96
TYR 128
ASP 218
TYR 228
LEU 266
ILE 214
PRO 219
PRO 227
GLY 240
GLY 239
LEU 238
ARG 283
ILE 230
GLU 220
HIS 99
GLY 262
CYS 263
SER 226
ARG 221
ASN 225
GLY 222
PHE 242
ILE 241
ILE 223
TYR 224
GLY 313
B
ILE 241
ARG 221
PHE 242
GLY 240
ASP 127
ILE 223
ASN 225
GLN 243
PRO 227
TYR 228
GLY 239
ARG 199
GLN 201
ASN 252
TYR 224
SER 226
LEU 215
ILE 214
ARG 283
ILE 230
LEU 238
GLU 220
HIS 99
ASP 218
GLY 222
TYR 55
GLY 313
THR 216
PRO 219
LEU 56
TYR 128
HIS 217
PHE 98
LEU 97
ASN 96
PRO 126
PHE 125
C
GLY 240
ARG 221
TYR 228
ILE 241
GLY 239
GLU 220
GLY 222
GLY 262
THR 258
ILE 214
ILE 202
PHE 242
PRO 227
LEU 238
PRO 219
CYS 263
TYR 224
ASN 225
THR 237
HIS 99
ASP 218
ILE 223
GLY 313
ARG 283
SER 226
PHE 98
HIS 217
TYR 55
LEU 97
ASN 96
THR 216
TYR 128
LEU 215
LEU 266

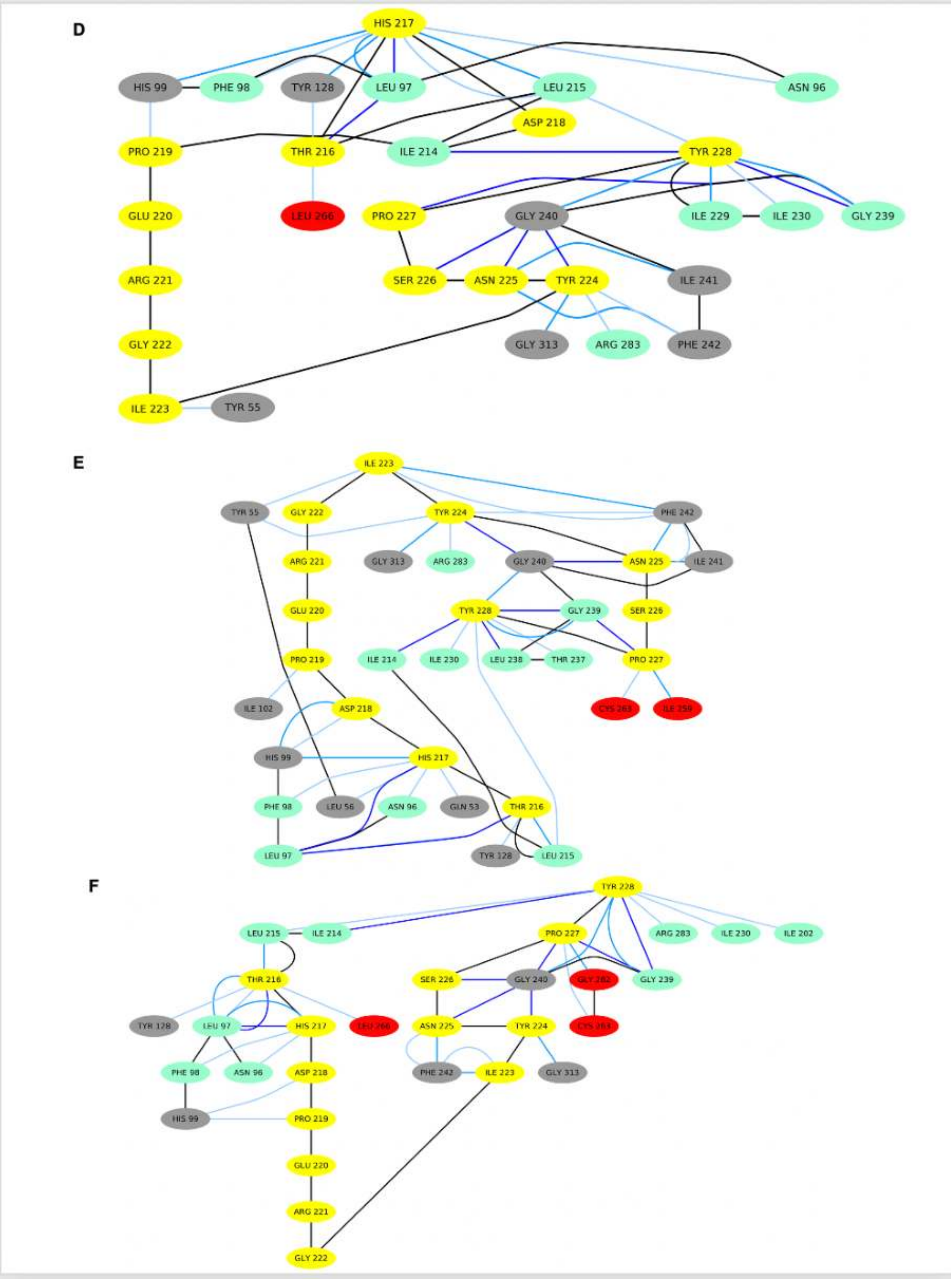
D
HIS 217
HIS 99
PHE 98
TYR 128
LEU 97
LEU 215
ASN 96
ASP 218
PRO 219
THR 216
ILE 214
TYR 228
LEU 266
GLU 220
PRO 227
GLY 240
ILE 229
ILE 230
GLY 239
SER 226
ASN 225
TYR 224
ILE 241
ARG 221
GLY 222
GLY 313
ARG 283
PHE 242
ILE 223
TYR 55
E
ILE 223
TYR 55
GLY 222
TYR 224
PHE 242
ARG 221
GLY 313
ARG 283
GLY 240
ASN 225
ILE 241
GLU 220
TYR 228
GLY 239
SER 226
PRO 219
ILE 214
ILE 230
LEU 238
THR 237
PRO 227
ILE 102
ASP 218
HIS 99
HIS 217
PHE 98
LEU 56
ASN 96
GLN 53
THR 216
LEU 97
TYR 128
LEU 215
F
TYR 228
LEU 215
ILE 214
PRO 227
ARG 283
ILE 230
ILE 202
THR 216
SER 226
GLY 240
GLY 239
TYR 128
LEU 97
HIS 217
ASN 225
TYR 224
PHE 98
ASN 96
ASP 218
PHE 242
ILE 223
GLY 313
HIS 99
PRO 219
GLU 220
ARG 221
GLY 222

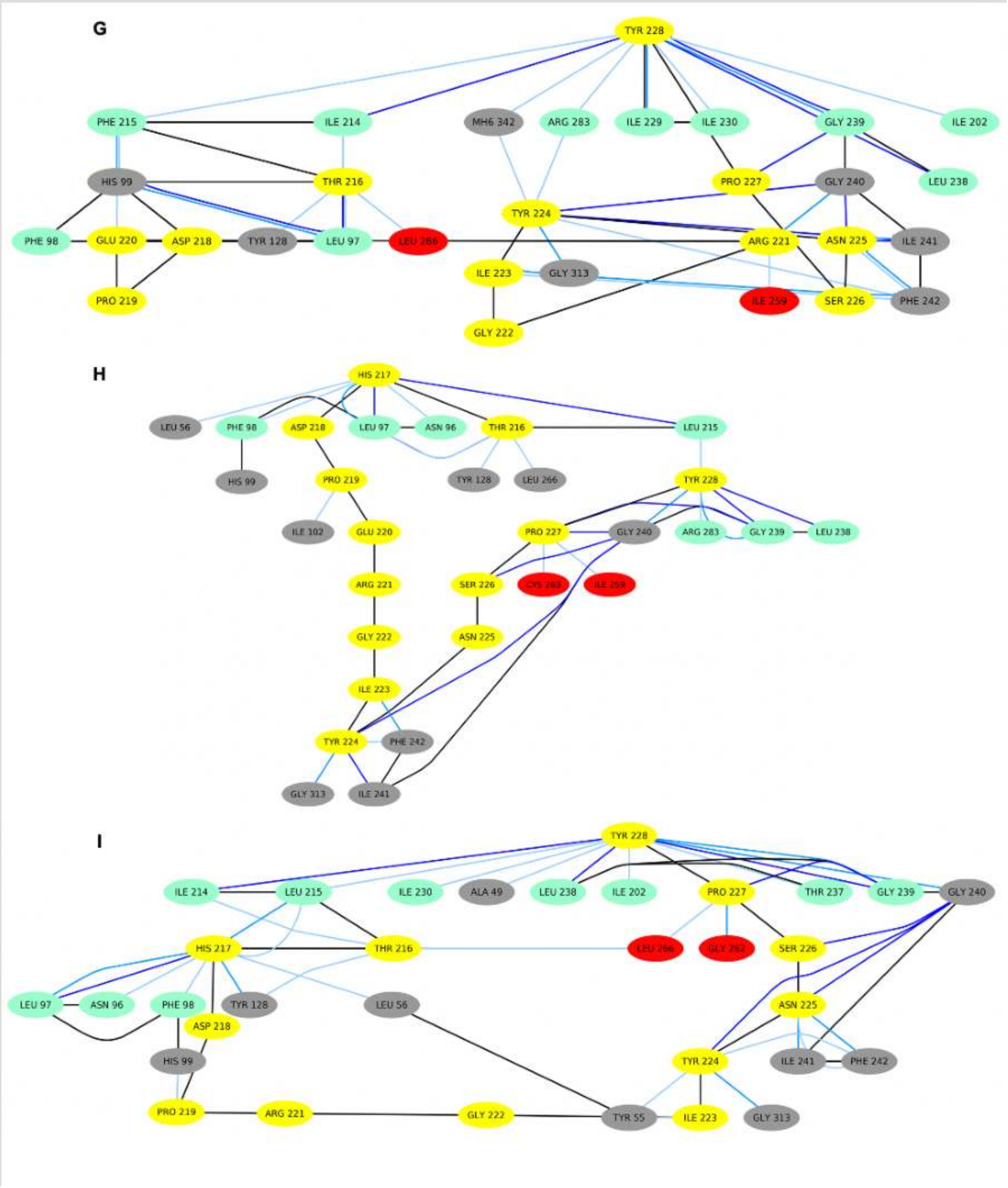
G
TYR 228
PHE 215
ILE 214
MH6 342
ARG 283
ILE 229
ILE 230
GLY 239
ILE 202
HIS 99
THR 216
PRO 227
GLY 240
LEU 238
TYR 224
PHE 98
GLU 220
ASP 218
TYR 128
LEU 97
LEU 266
ARG 221
ASN 225
ILE 241
ILE 223
GLY 313
ILE 259
SER 226
PHE 242
PRO 219
GLY 222
H
HIS 217
LEU 56
PHE 98
ASP 218
LEU 97
ASN 96
THR 216
LEU 215
HIS 99
PRO 219
TYR 128
LEU 266
TYR 228
ILE 102
GLU 220
PRO 227
GLY 240
ARG 283
GLY 239
LEU 238
ARG 221
SER 226
ILE 259
GLY 222
ASN 225
ILE 223
TYR 224
PHE 242
GLY 313
ILE 241
I
TYR 228
ILE 214
LEU 215
ILE 230
ALA 49
LEU 238
ILE 202
PRO 227
THR 237
GLY 239
GLY 240
HIS 217
THR 216
LEU 266
SER 226
LEU 97
ASN 96
PHE 98
TYR 128
LEU 56
ASN 225
ASP 218
HIS 99
TYR 224
ILE 241
PHE 242
PRO 219
ARG 221
GLY 222
TYR 55
ILE 223
GLY 313

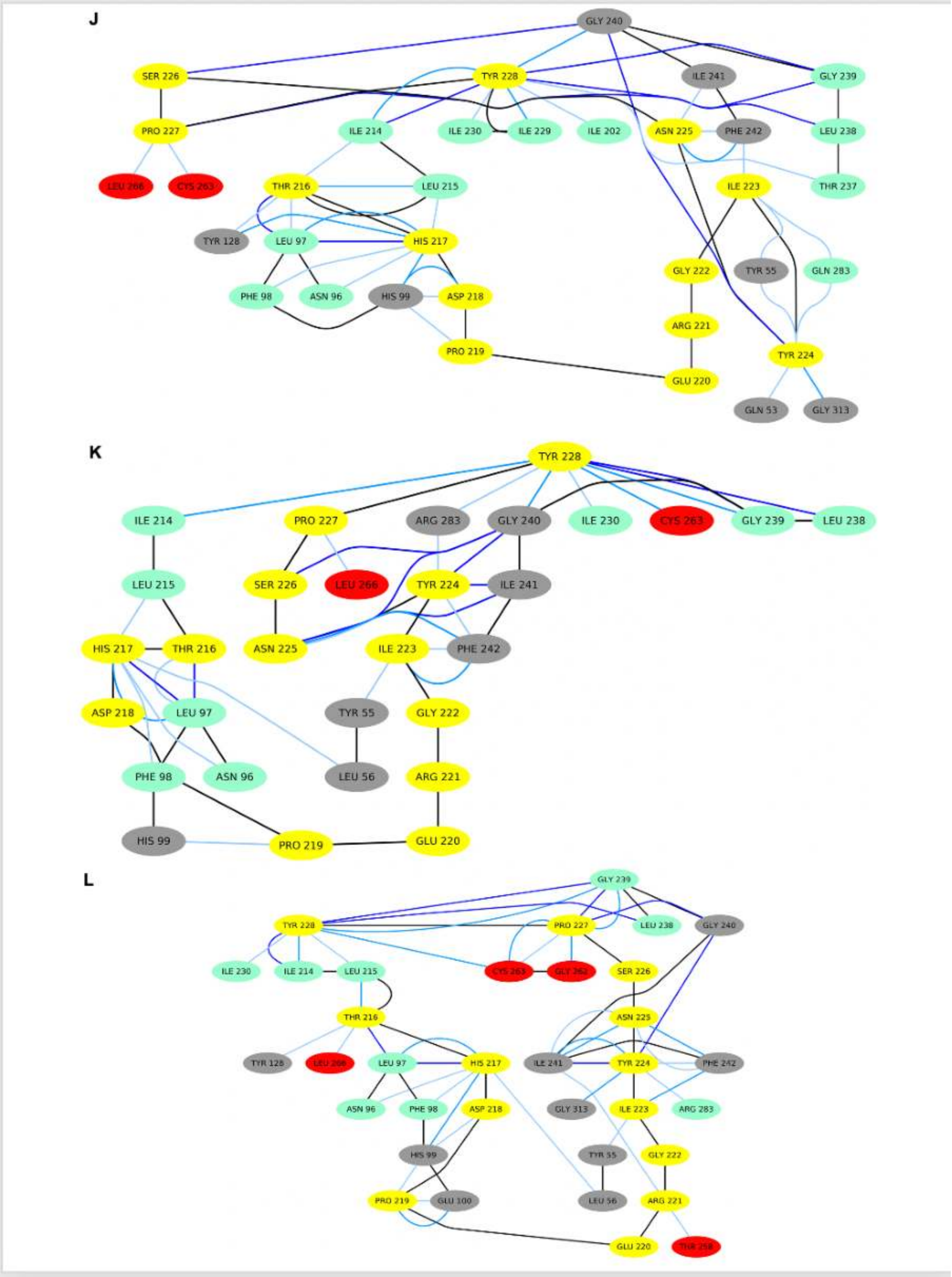
J
GLY 240
SER 226
TYR 228
ILE 241
GLY 239
PRO 227
ILE 214
ILE 230
ILE 229
ILE 202
ASN 225
PHE 242
LEU 238
LEU 266
CYS 263
THR 216
LEU 215
ILE 223
THR 237
TYR 128
LEU 97
HIS 217
GLY 222
TYR 55
GLN 283
PHE 98
ASN 96
HIS 99
ASP 218
ARG 221
PRO 219
TYR 224
GLU 220
GLN 53
GLY 313
K
TYR 228
ILE 214
PRO 227
ARG 283
GLY 240
ILE 230
CYS 263
GLY 239
LEU 238
LEU 215
SER 226
LEU 266
TYR 224
ILE 241
HIS 217
THR 216
ASN 225
ILE 223
PHE 242
ASP 218
LEU 97
TYR 55
GLY 222
PHE 98
ASN 96
LEU 56
ARG 221
HIS 99
PRO 219
GLU 220
L
GLY 239
TYR 228
PRO 227
LEU 238
GLY 240
ILE 230
ILE 214
LEU 215
CYS 263
SER 226
THR 216
ASN 225
TYR 128
LEU 266
LEU 97
HIS 217
ILE 241
TYR 224
PHE 242
ASN 96
PHE 98
ASP 218
GLY 313
ILE 223
ARG 283
HIS 99
TYR 55
GLY 222
PRO 219
GLU 100
LEU 56
ARG 221
GLU 220

M

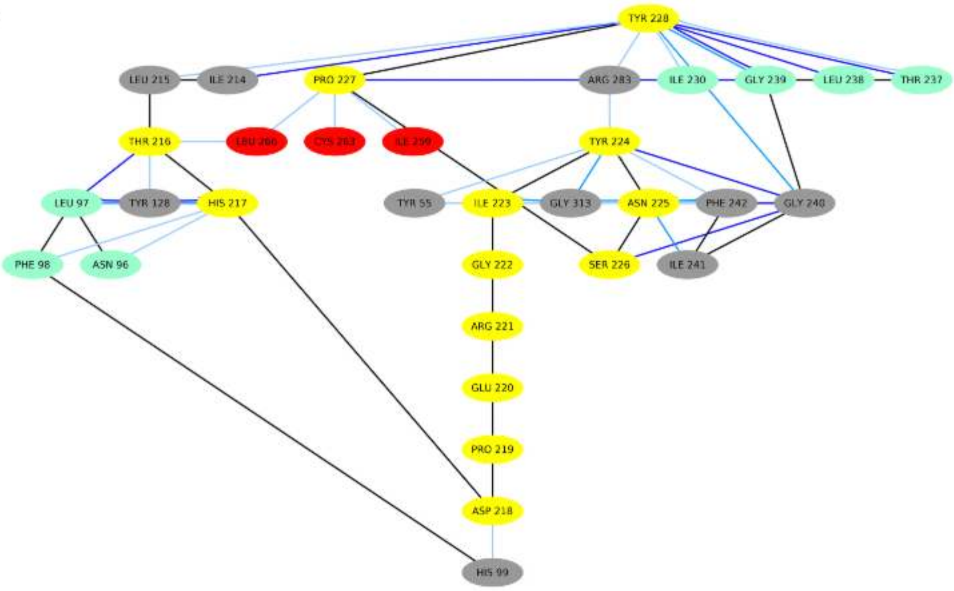

Figure S10. Residue interaction networks between wild-type and rare DAAO variants for the active site loop comprising residues 216-228. Comparison of residue interaction networks between (A-M) for variants (V5A, H78Y, F90V, P103L, R115W, P119L, L215F, P268S, R279Q, R283Q, R286C, L329F, G331E) highlighting the changes in network interaction for active site loop comprising residues 216-228. Amino acids are represented as nodes and interaction types are represented as edges. Blue color edge denotes the hydrogen-bonds and black color edge denotes the contact between the residues. Mutated and active site residues are shown in yellow colored nodes and the remaining nodes are shown according to their secondary structure type; helix: red, sheet: light green, and loop: gray color.

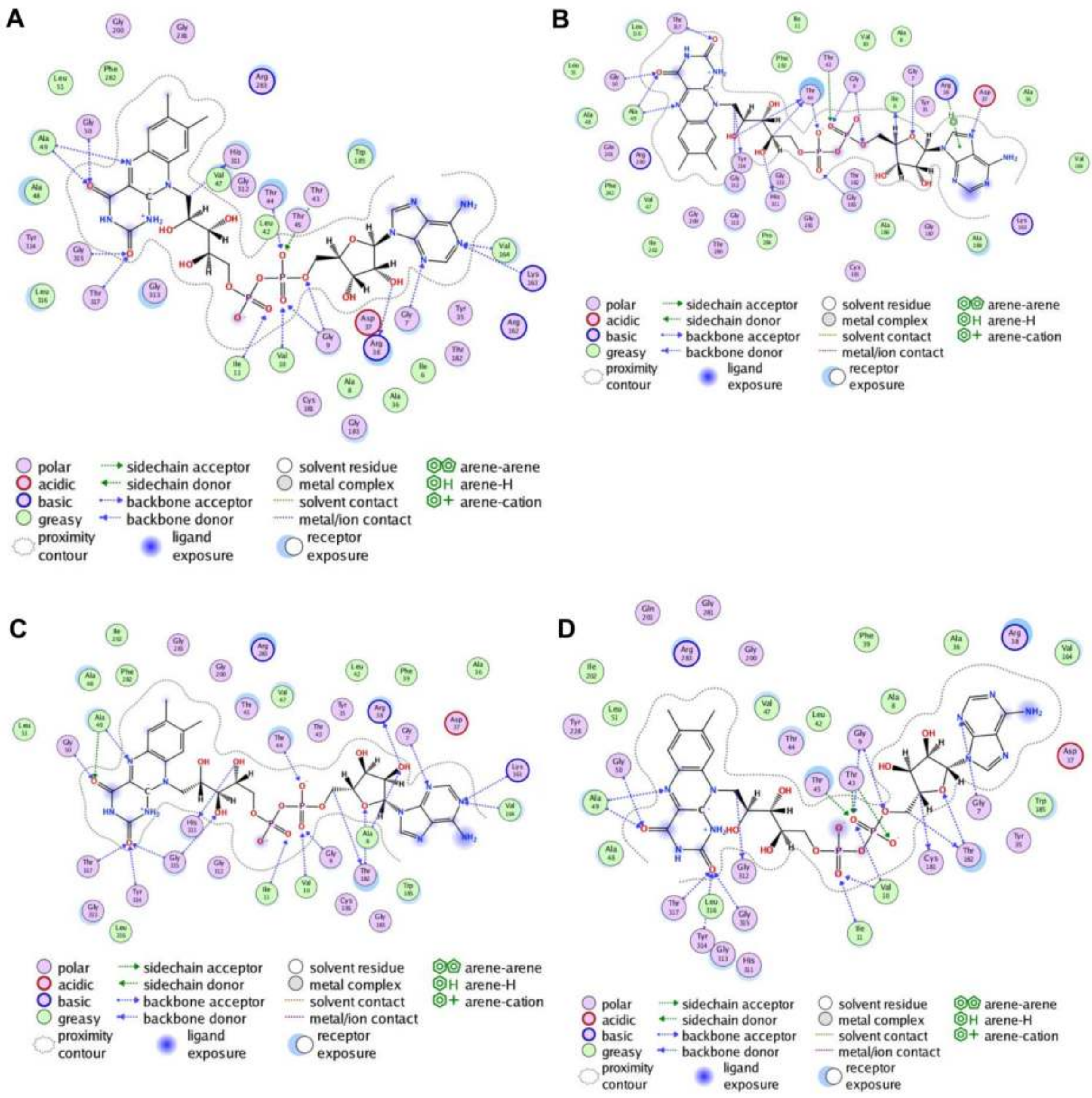

Figure S11. Molecular interaction of FAD with rare DAAO variants. Two-dimensional ligand interaction diagrams of (A) L215F, (B) D46N, (C) S340F and (D) R279Q variants showing the perturbed interactions in L215F and D46N as compared to S340F and R279Q variants. FAD is shown as stick model and nodes denote the amino acid residues. The molecular interaction types are presented as a legend to the figure.

Table S1. The differences in residue-residue interactions for the rare variants in relation to the active site residues.

D46N	S340F	V5A	H78Y	F90V	P103L	R115W	P119L	L215F	R279Q	R283Q	R286C	L329F	G331E
Asn46-Ala48	Phe340-Glu335	Ala5-Val34	Tyr78-Leu76	Val90-Leu89	Leu103-Asp104	Trp115-Lys116	Leu119-Leu122	Phe215-Phe213	Gln279-Thr280	Gln283-Val285	Cys286-Val285	Phe329-Lys328	Glu331-Lys328
Asn46-Ile202	Phe340-Lys338	Ala5-Lys33	Tyr78-Leu75	Val90-Ile138	Leu103-Ile102	Trp115-Trp132		Phe215-His217	Gln279-Val47	Gln283-Gly312	Cys286-Pro287	Phe329-Arg332	Glu331-Leu334
Asn46-Lys142	Phe340-Leu339	Ala5-Val179	Tyr78-Tyr74	Val90-Leu89		Trp115-Gly113		Phe215-Leu97	Gln279-Asn251	Gln283-Ile223	Cys286-Gln288	Phe329-Glu325	Glu331-Ile333
Asn46-Thr44	Phe340-Val26	Ala5-Val3	Tyr78-His80			Trp115-Phe133		Phe215-Gln53	Gln279-Glu278		Cys286-Gly310	Phe329-Leu291	Glu331-Phe330
Asn46-Thr43			Tyr78-Ser81					Phe215-Ile214	Gln279-Ile202			Phe329-Ile333	Glu331-Tyr23
Asn46-Thr40			Tyr78-Leu87					Phe215-Asn96				Phe329-Ala326	
Asn46-Pro41								Phe215-Tyr95					
